# Interactive histogenesis of axonal strata and proliferative zones in the human fetal cerebral wall

**DOI:** 10.1007/s00429-018-1721-2

**Published:** 2018-08-09

**Authors:** Iris Žunić Išasegi, Milan Radoš, Željka Krsnik, Marko Radoš, Vesna Benjak, Ivica Kostović

**Affiliations:** 1 Croatian Institute for Brain Research, Centar of Research Excellence for Basic, Clinical and Translational Neuroscience, University of Zagreb, School of Medicine, Zagreb, Croatia; 2 Department of Radiology, Clinical Hospital Center Zagreb, University of Zagreb, School of Medicine, Zagreb, Croatia; 3 Department of Pediatrics, Clinical Hospital Center Zagreb, University of Zagreb, School of Medicine, Zagreb, Croatia

**Keywords:** Sagittal axonal strata, Fetal brain, Proliferative and migratory neurons, Glia, White matter integrity

## Abstract

Development of the cerebral wall is characterized by partially overlapping histogenetic events. However, little is known with regards to when, where, and how growing axonal pathways interact with progenitor cell lineages in the proliferative zones of the human fetal cerebrum. We analyzed the developmental continuity and spatial distribution of the axonal sagittal strata (SS) and their relationship with proliferative zones in a series of human brains (8–40 post-conceptional weeks; PCW) by comparing histological, histochemical, and immunocytochemical data with magnetic resonance imaging (MRI). Between 8.5 and 11 PCW, thalamocortical fibers from the intermediate zone (IZ) were initially dispersed throughout the subventricular zone (SVZ), while sizeable axonal “invasion” occurred between 12.5 and 15 PCW followed by callosal fibers which “delaminated” the ventricular zone-inner SVZ from the outer SVZ (OSVZ). During midgestation, the SS extensively invaded the OSVZ, separating cell bands, and a new multilaminar axonal-cellular compartment (MACC) was formed. Preterm period reveals increased complexity of the MACC in terms of glial architecture and the thinning of proliferative bands. The addition of associative fibers and the formation of the centrum semiovale separated the SS from the subplate. In vivo MRI of the occipital SS indicates a “triplet” structure of alternating hypointense and hyperintense bands. Our results highlighted the developmental continuity of sagittally oriented “corridors” of projection, commissural and associative fibers, and histogenetic interaction with progenitors, neurons, and glia. Histogenetical changes in the MACC, and consequently, delineation of the SS on MRI, may serve as a relevant indicator of white matter microstructural integrity in the developing brain.

## Introduction

One of the remarkable developmental features of the human brain is its punctual timetable with respect to the sequential growth of different classes of axonal pathways which become precisely arranged within the cerebral wall, forming complex geometric white matter structure by the time of birth. The complexity of the adult white matter reflects the spatial arrangement and connectivity of projection, along with the commissural and associative pathways which are composed mainly of myelinated axons in layers III, V, and VI of the principal neurons in the neocortex and the massive thalamocortical projection system (Jones 1987; Schmahmann and Pandya 2006). The organization of cerebral white matter has been extensively investigated in classical neuroanatomical studies. Some classical studies of the human brain focused on the origin, trajectory, and termination of pathways, while other studies focused on the segmentation and geometry of the fiber system (Sachs 1892; Déjerine 1895; Von Monakow 1905; Brodmann 1914; Flechsig 1920; Polyak 1932). Modern neuroimaging and neuroanatomical studies have allowed us to extend our knowledge of the segmentation and geometry of the white matter pathways and have provided a better visualization and three-dimensional (3D) reconstructions of white matter (Makris et al. 1999, 2007; Fischl et al. 2002; Mori and van Zijl 2002; Tamura et al. 2003; Maas et al. 2004; Schmahmann and Pandya 2006, 2007; Huang et al. 2006; Bassi et al. 2008; Catani and Thiebaut de Schotten 2008; Axer et al. 2011; Wedeen et al. 2012; Zilles et al. 2016).

The most recent approach to studying white matter connectivity is based upon whole-brain magnetic resonance imaging (MRI) and the analysis of structural white matter networks constructed using the tools of graph theory (Sporns et al. 2005; Hagmann et al. 2008; Collin and Van Den Heuvel 2013; Tymofiyeva et al. 2014; Cao et al. 2017). Despite growing evidence in support of the specific organization of white matter, there have been very few attempts to classify white matter in characteristic divisions which would correspond to the segmentation described in classical literature (Makris et al. 1999; Fischl et al. 2002). The classical description of five white matter segments (Sachs 1892; Déjerine 1895; Von Monakow 1905; Brodmann 1914), spanning from ventricle to cortex, which was originally described in the developmental literature (Judaš et al. 2005), is still not widely accepted by the current neuroimaging studies. The five segments of white matter (I—callosum with periventricular fibers; II—crossroads and sagittal strata; III—centrum semiovale; IV—gyral white matter; V—intracortical white matter; Kostović et al. 2014a) contain different classes of projection, associative, and commissural pathways. From a developmental point of view, it is very important to understand how different classes of axons grow within expanding hemispheres, how they become incorporated into white matter segments, and how the laminar intermediate zone (fetal “white” matter) transforms into adult pattern of white matter radial segmentation. Adopting a developmental approach has an additional advantage in that the geometrical arrangement of the fiber system is more transparent in the developing human brain compared to the adult situation (Kostović et al. 2002a; Vasung et al. 2010) and is, therefore, more accessible for reconstruction. Analyzing the sequential development of white matter segments also provides better insight of the sequential growth of different classes of axons (Kostović and Judaš 2010; Vasung et al. 2010; Dubois et al. 2014, 2015; Kostović et al. 2014b). In developmental studies of growing cerebral pathways, it is possible to follow pathways from the site of origin via growth trajectories to their distribution in the target area (Kostović and Goldman-Rakić 1983; Kostović and Rakić 1984; Kostović 1986; Vasung et al. 2010).

The rationale behind the present research is that changes in the structure and development of the sagittal strata (SS) will not only show growth “corridors” and the trajectories of major axonal pathways but will also reflect dynamic histogenetic changes such as proliferation and synaptogenesis. To be more specific, the axonal SS run at a strategic depth within the cerebral wall, situated between the transient ventricular–subventricular proliferative zones on one side, and the synaptic subplate (SP) compartment on the pial (superficial) side. In addition, in contrast to an internal capsule (IC), which has been investigated more frequently, the SS contain not only projection, but also associative pathways. Since major compartments in the cerebral wall can be visualized on both in vitro and in vivo MRI images (Kostović et al. 2002a; Maas et al. 2004; Huang et al. 2006; Radoš et al. 2006; Kasprian et al. 2008; Widjaja et al. 2010; Corbett-Detig et al. 2011; Huang and Vasung 2014; Xu et al. 2014; Wang et al. 2015; Vasung et al. 2016), histological analysis of the development of the main axonal strata within the cerebral compartments will provide us with useful information relating MR images to the spatio-temporal parameters of axonal growth in the human cerebrum. Furthermore, analysis of the stratification and sublaminar distribution of fibers within the axonal SS will provide insight relating to the condition of different fiber classes, thus providing us with the opportunity to use these normative data to evaluate the integrity of white matter in preterm infants and help to develop additional structural criteria for the analysis of perinatal white matter lesions (Volpe 2009; Kidokoro et al. 2011; Kostović et al. 2014b).

## Materials and methods

We used proliferative, neuronal, and glial markers to visualize developmental and spatial relationships of the growing axonal SS, along with cellular and fibrillar indicators of other neurogenetic and gliogenetic events in the human cerebral wall. Human post-mortem brain tissue was collected from 24 brains at the early fetal stage, from approximately 9 post-conceptional weeks (PCW) to newborn, without macroscopical or microscopical pathological changes. These specimens were processed and analyzed during the histological part of the study. These brain specimens were part of the Zagreb Neuroembryological Collection (Kostović et al. 1991; Judaš et al. 2011) and were obtained during regular autopsies after either medically indicated or spontaneous abortions, or following the death of infants who were born at term or prematurely, at a number of clinical hospitals affiliated to the University of Zagreb, School of Medicine. Sampling of brain tissue was performed in accordance with the Declaration of Helsinki 2000, and was approved by the Internal Review Board of the Ethical Committee of the University of Zagreb, School of Medicine.

Fetal age was estimated on the basis of crown-rump length (CRL, in millimeters) and pregnancy records, and was expressed as PCW. The MRI part of the study included scans of 10 fetuses in utero (12–32 PCW, scanned as a result of maternal indications), 13 infants born prematurely (birth age ranging from 26 to 32 PCW) who were first scanned shortly after birth, and then again at term equivalent age (TEA), and 5 control infants born and scanned at term (due to extracranial indications). Our initial cohort of prematurely born children consisted of 43 infants, but, in our current study group, we included only normotypic children, which implies that they did not have any structural pathological changes on MRI or neurological deficits in their anamnesis (see our previous paper Kostović et al. 2014b). In each case, we obtained informed consent (parents, legal guardian, or participants themselves in cases of scanning pregnant woman) for MRI scanning, and all examinations were controlled and approved by the Institutional Review Board of the University of Zagreb, School of Medicine. In addition, we carried out MRI scans in vitro on 25 post-mortem brains (age ranging from 11 to 35 PCW) from the Zagreb Brain Collection (http://www.zagrebbraincollection.hr/index.php). Histological images were compared with post-mortem diffusion tensor imaging (DTI)-based fiber tractography from the Maryland Brain and Tissue Bank (contracts No NO1-HD-4-3368 and NO1-HD-4-3383).

### MRI protocol

This study used a 1.5T MRI scanner (Symphony, Siemens) and a 3T MRI scanner (Magnetom Prisma^FIT,^ Siemens) for in vivo and in vitro MR imaging. MRI scans of fetuses, scanned in utero, were obtained using a 1.5T MRI scanner during medically indicated diagnostic examinations of the pelvic region in pregnant woman, or due to their own extracranial medical indications. Only fetuses with no signs of brain pathology were included in this study. A six-element body coil was used to acquire fetal MRI scans using the T2-weighted half-Fourier single shot turbo spin-echo (HASTE) sequence (TR/TE = 1400 ms/96 ms, FOV = 380 × 310 mm, resolution = 384 × 280, voxel size = 1 × 1 × 3 mm; duration = 1:10 min). Postnatal subjects were divided in two groups. The first group included infants who were born prematurely and were scanned shortly after birth on a 1.5T MRI scanner in the Clinical Hospital of the University of Zagreb (T2-weighted turbo spin-echo, TR/TE = 5050 ms/116 ms, FOV = 200 × 104 mm; resolution = 448 × 152; voxel size = 0.5 × 0.6 × 5 mm; duration 2:46 min), and then again at TEA on 3T MRI scanner (T2-weighted turbo spin-echo TR/TE = 6000 ms/96 ms, FOV = 220 × 96 mm; resolution = 512 × 179; voxel size = 0.4 × 0.4 × 1.5 mm; duration 3:30 min). The second group consisted of normal children who were born at term and were scanned on a 3T MRI scanner as a result of extracranial indications (using the same sequences as for premature infants). Post-mortem human fetal brains were scanned on a 3T MRI scanner [T1-weighted high-resolution magnetization-prepared rapid acquisition gradient-echo (MPRAGE) sequences] (TR/TE = 14 ms/4.6 ms; FOV = 125 × 90 mm; resolution = 512 × 368; voxel size = 0.25 × 0.25 × 0.30 mm; duration 2:48 h).

### Histological and immunocytochemical protocol

Post-mortem brains were sliced into blocks and fixed in 4% paraformaldehyde in 0.1M phosphate-buffered saline, PBS, pH 7.4. Tissue blocks were then embedded in paraffin wax, and sections prepared using a microtome at 20-µm thickness. Classical histological Cresyl violet (Nissl) or histochemical (Achetylcholinesterase, AChE) staining was then performed as described previously (Kostović and Goldman-Rakić 1983). Immunohistochemistry was performed following deparaffinization of the paraffin-embedded blocks. In brief, sections were treated with 0.3% hydrogen peroxide, incubated in blocking solution (3% bovine serum albumine, BSA, and 0.5% Triton x-100, Sigma, St. Louis, MO, USA) in 0.1M PBS, and incubated overnight with primary antibodies at room temperature. We used following primary antibodies to identify different cell types such as, neurons (anti-NeuN, Abcam, ab104225, 1:1000-not shown), radial glia (anti-Vimentin, DAKO, m-7020 1:100), glial cells (anti-GFAP, DAKO, z-0334, 1:1000), microglia (anti-CD45, Abcam, ab10559, 1 µg/ml; anti-Iba1, Wako Chemicals, 019.19741, 1:1000), oligodendrocytes (anti-Olig2, IBL, 0.5 µg/ml), proliferative cells (anti-Ki67, DAKO, m-7240, 1:100; Thermo Fisher Sc. PA5-16446, 1:100; anti-Pax6, Abcam, ab5790, 1:75; anti-Sox2, Santa Cruz, sc-365823, 1:250), indicators of histogenetic events, such as myelination marker (anti-SMI 99 Biolegend; 808401, 1:1000), and synaptogenesis markers (anti-SNAP-25, Biolegend, 836301, 1:1000; anti-synaptophysin, DAKO, m-7315, 1:100-not shown). Corresponding secondary biotinylated antibodies (Vectastain ABC kit, Vector Laboratories, Burlingame, CA, USA) were subsequently applied in accordance with the manufacturer’s protocol. Finally, positive staining was visualized using 3,3-diaminobenzidine with a metal enhancer in accordance with the manufacturer’s protocol (Sigma, St. Louis, MO), and sections were mounted and coverslipped (Histamount, National Diagnostics, Charlotte, NC). Images were acquired using a high-resolution digital slide scanner NanoZoomer 2.0RS (Hamamatsu, Japan) and images prepared using Microsoft Publisher (Microsoft Office 2016).

## Results

### During the early fetal period (8-11PCW), the first fibers from the thalamus and basal forebrain ran sagittally through the expanding intermediate zone and initially dispersed the subventricular zone

**Fig. 1:**
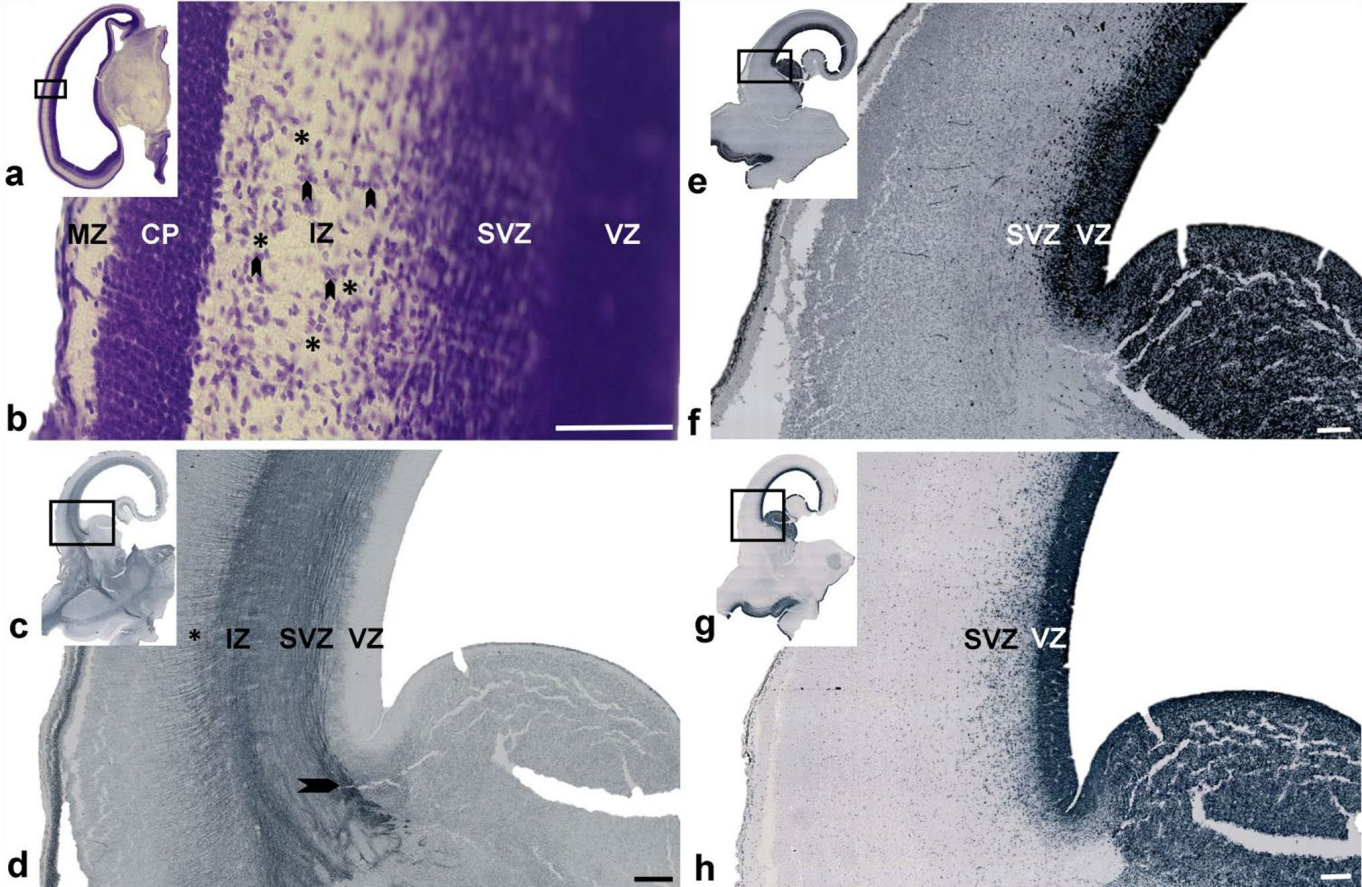
Early sagittal fibers on coronal Nissl (**a, b**) and immunostained histological sections (**c**–**h**) through the occipital (**a, b**) and midlateral levels (**c**–**h**) of the brain at 9 (**a, b**) and 11 (**c**–**h**) PCW. The rectangle in ‘a’, ‘c’, ‘e’, and ‘g’ are shown at higher magnifications in ‘b’, ‘d’, ‘f’, and ‘h’ respectively. The intermediate zone (IZ) contains large fiber bundles (**b** asterix), running in a sagittal direction separated by groups of migratory neurons (**b** arrowheads). Coronal sections through the brain at 11 PCW showed SNAP25 immuno-positivity to sagittal fibers in the IZ (**c, d**). Some fibers originating from the thalamus showed radial orientation before attaining sagittal orientation (**d** arrowhead). The IZ is well delineated from the presubplate (**d** asterisk) and ventricular zone (VZ), but intermingles with the subventricular zone (SVZ). Staining of the same brain (11 PCW) for SOX2 (**e, f**) and Ki67 (**g, h**) showing a homogenous VZ but dispersed SVZ. (*CP* cortical plate; *MZ* marginal zone). Scale bar = 100 µm (**b**); 200 µm (**d, f**)

Analysis of serial sections through the telencephalon in the youngest group examined (8.5–11 PCW) revealed a well-developed intermediate zone (IZ), situated between the subventricular zone (SVZ), and the presubplate and cortical plate (CP) compartments (Fig. 1). On Nissl-stained sections, the IZ appeared pale; this was due to the richness of fibers and paucity of cell bodies. Fibers in the occipital and frontal portions of the cerebral hemisphere ran in an antero-posterior sagittal direction. Large bundles of cross-sectioned fibers (Fig. 1b, asterix) were separated by strands of migratory neurons which were oriented in a radial direction, respectively, from ventricle to pia (Fig. 1b, arrowheads).

In contrast to the sagittal orientation of fibers in the frontal and occipital cortex, the midlateral regions of the telencephalon contained radially oriented fiber bundles (Fig. 1c, d) which originated from the thalamus, and were directed in a fan-like radiation to the midlateral telencephalon (not shown, but described in our previous publications; Kostović and Goldman-Rakic 1983; Kostovic and Rakic 1984; Krsnik et al. 2017).

At this early fetal age, the SVZ is not “delaminated” from the ventricular zone (VZ), as seen on Nissl-stained-thick celloidin or paraffin sections (Fig. 1a, b), and the distinction of these two proliferative zones was transparent only on very thin sections or on sections stained immunohistochemically for axons. Specifically, fibers entering the IZ showed transient SNAP25 immunoreactivity (Fig. 1c, d). On sections which were immunostained for SNAP25, the external boundary of the IZ (Fig. 1c, IZ) was sharply delineated from the presubplate (Fig. 1d, asterisk) and the VZ stood out as a non-fibrillar layer. The border between sagittal fibers of the IZ and SVZ was not too evident due to the fact that sagittal fibers already ran through the external superficial portion of the SVZ causing the initial stratification of the SVZ (Fig. 1d). Consequently, SVZ was not homogenous on sections immunostained for the neuroepithelial progenitor cell marker SOX2 (Fig. 1e, f) or the general proliferative marker Ki67 (Fig. 1g, h); however, the VZ was well delineated and homogenous (Fig. 1).

**Fig. 2:**
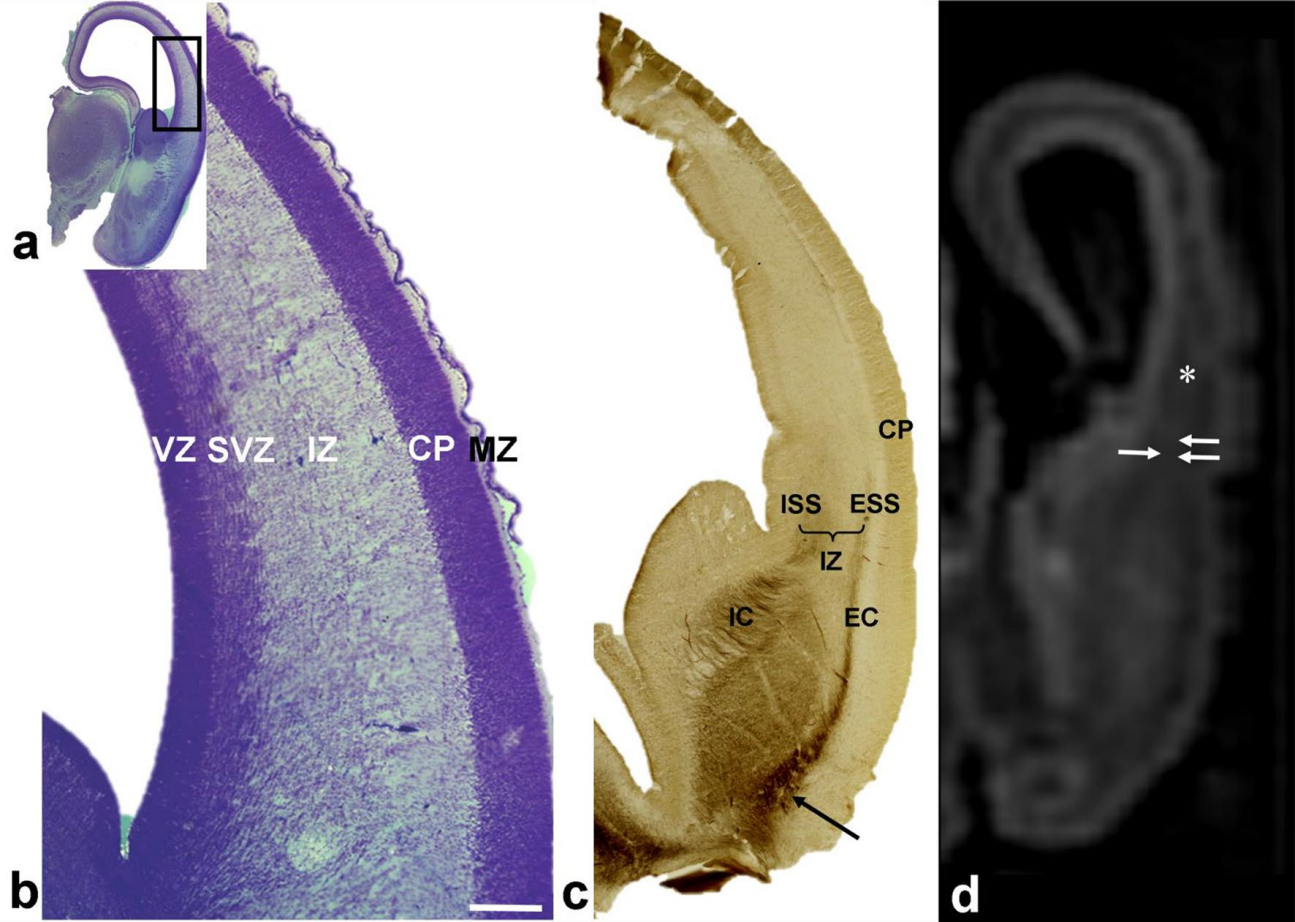
Increasing thickness of the intermediate zone (IZ) and the contribution of AchE-reactive thalamic fibers in 10.5 PCW old brain (**a**–**c**). Initial axonal sagittal strata develop on the deep and superficial border of the intermediate zone (**b, d** IZ; **e** asterisk). ISS partially overlap with SVZ. Rectangle in ‘**a**’ shown at higher magnifications in ‘**b**’. AChE staining **c** shows two AChE-stained strata: the internal stratum (**c** ISS), in connection with the internal capsule (IC), and the external stratum (**c** ESS) in continuity with the external capsule (EC) and the basal forebrain (**c** arrow). In vitro T1-weighted MRI of fetal post-mortem brain at 12 PCW (coronal plane; **e**) showing trilaminar organization. Points of exit for the internal capsule (**e** one arrow) and external capsule (**e** two arrows) indicate the approximate depth of the ISS and ESS. (*VZ* ventricular zone; *SVZ* subventricular zone; *CP* cortical plate; *MZ* marginal zone). Scale bar = 200 µm

In older specimens from this period, there was an increase in the thickness of the IZ (Fig. 2a, b). For the first time, during this phase, it was possible to identify fibers entering in longitudinal and sagittal directions of the IZ and visualize the first division of sagittally running fibers into the deep internal sagittal stratum (ISS) and the superficial external sagittal stratum (ESS) which corresponds to the primordial external capsule (EC) (Fig. 2c). AChE staining of growing thalamic and basal forebrain fibers (Kostović and Rakić 1984; Kostović 1986) revealed that fibers of the ISS ran into the IC and further into the thalamus. Fibers from the ESS were followed into the AChE-reactive basal forebrain (Fig. 2c, arrow). Due to the growth of the callosal fiber system after 10.5 PCW (see Rakic and Yakovlev 1968), we were able to visualize the initial “delamination” of the VZ from the SVZ, into the medial neocortical areas, which were invaded by the initial growth of the corpus callosum (CC).

In vitro MRI scanning (Fig. 2d) of corresponding developmental phases showed basic trilaminar organization (Radoš et al. 2006; see also the three-layer organization described by; Huang et al. 2013), in which the internal lamina (towards the ventricle) was cell dense proliferative VZ/SVZ, the external lamina (towards the pia) was cell dense CP, and the intermediate lamina corresponds to the IZ with sagittal and tangential SS. It is important to note that, on the conventional in vitro MRI, one cannot visualize the border between the SVZ and IZ. Consequently, the exact position of the initial ISS and ESS was not discernible within the IZ. However, by following the exit of the IC (internally) and the external border of the striatum (externally), it was possible to estimate the approximate subpial depth of the initial ISS (Fig. 2d, one arrow) and ESS (Fig. 2d, two arrows), even using in vitro MRI images.

### The beginning of the mid-fetal period (12.5–15 PCW): fibers from the axonal SS and CC invaded the proliferative zones

**Fig. 3:**
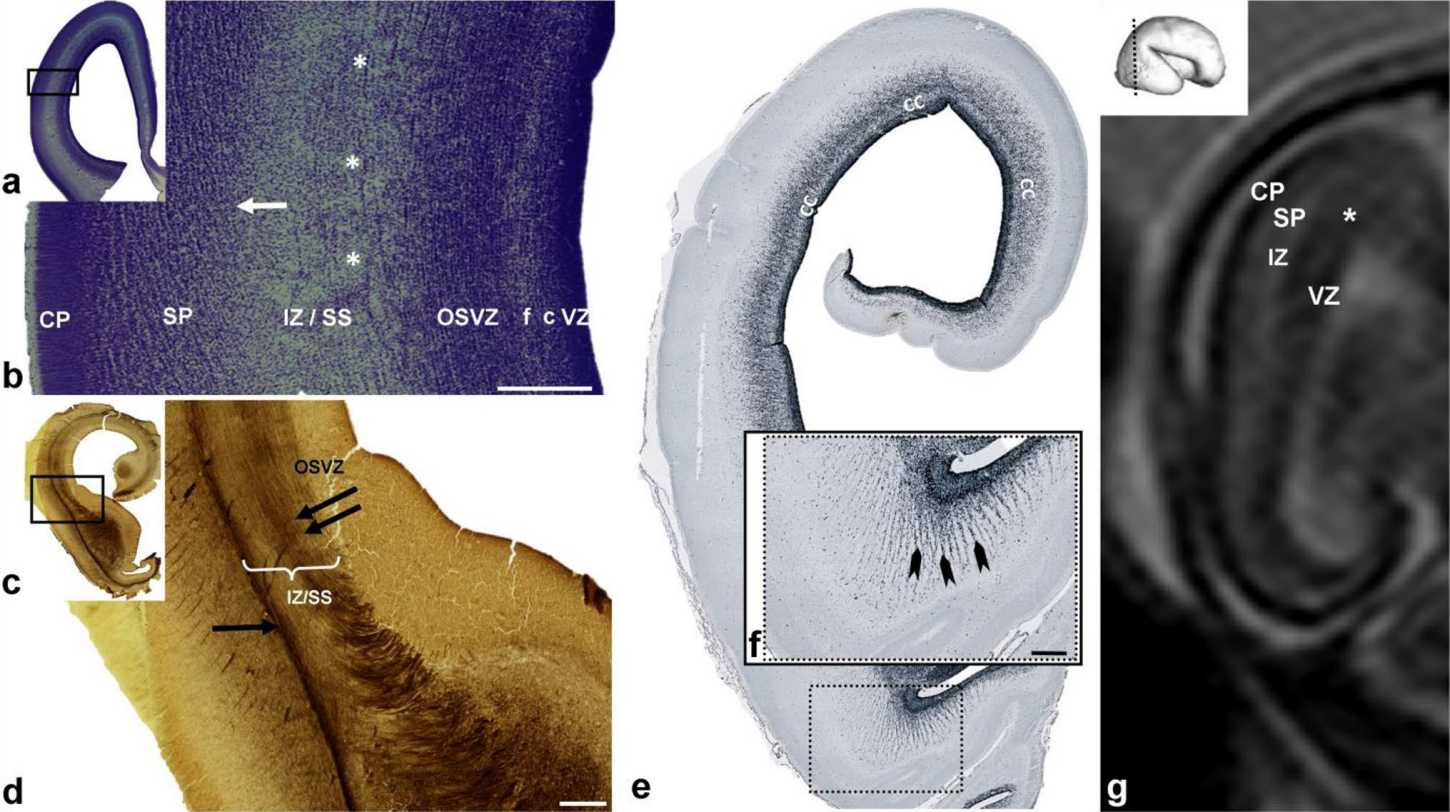
Fibers from the axonal sagittal strata (SS) participate in subplate (SP) formation. Secondary expansion of the SP from the deep loose portion of the “second” cortical plate (CP) is clearly visible on Nissl-stained sections of brain at 14.5 PCW (**a, b**), as shown on a coronal section through the frontal lobe. Rectangles in **a, c, e** are shown at higher magnifications in **b, d, f**, respectively. The border between the intermediate zone (IZ) and the SP is indicated by an arrow (**b**), and large fiber bundles are marked by an asterix (**b**). AChE staining of coronal sections through brain at 13.5 PCW (**c, d**) shows how AChE-reactive fibers from the thalamus-internal capsule (**d** double arrows) partially ran through the outer subventricular zone (OSVZ) as well as the AChE-reactive external SS (**d** one arrow). Relationship of the fiber system rising to sagittal strata and proliferative cerebral zones in a 13 PCW brain shown on section immunostained for the proliferative marker Ki67 (**e, f**). Massive fibers from the anterior limb of the internal capsule (**f** arrowheads) penetrate through ganglionic eminence and the subventricular zone. Sagittal continuation of callosal fibers (unstained AChE band) runs through the deepest portion of the SVZ (**e** CC). T2-haste MRI of brain at 12 PCW in utero (**g**) showing subplate formation (SP), which became strategically positioned between the IZ  and the cortical plate (CP), leaving more space for the ingrowth of fibers from the sagittal fiber compartment of IZ. (**e** asterisk-crossroad of fibers; *VZ* ventricular zone, *f* fiber-rich subventricular zone, *c* cell-rich subventricular zone). Scale bar = 500 µm

During this restricted period, massive sagittal thalamocortical and cortico-subcortical fibers, emanating from the IC, penetrated through ganglionic eminence and the SVZ, intermingling with proliferative cells (Fig. 3f). At the points where fibers showed radial orientation, we observed that proliferative neurons formed radially oriented strands (Fig. 3f, arrowheads). On more frontal and occipital regions, where fibers showed sagittal orientation, proliferative neurons were dispersed in sublaminas (Fig. 3b). During this period, sagittally running callosal fibers (Fig. 3e, CC) began to penetrate through the deepest portion of the SVZ, adjacent to the VZ, splitting the SVZ into inner and regularly serrated SVZ, juxtaposed to the VZ, and a larger, proper outer SVZ (OSVZ). The periventricular fibers, running between inner SVZ and OSVZ, were described previously by our group (Kostović et al. 2002a), and correspond to the inner fibrillar layer by Smart et al. 2002, and GAP43 and SRGP1 reactive periventricular system described by Molnár and Clowry (2012) in a 15 PCW old brain. We named the splitting of proliferative zones into a deep ventricular contingent (VZ and inner SVZ) and a more superficial contingent, as “delamination”. In current neuroembryological literature, this term is used to describe the process in which neural precursors leave the VZ and become intermediate progenitors (Arai and Taverna 2017).

The expansion of sagittally and tangentially directed fibers (axonal SS) from the IZ also occured on its own superficial (pial) side, where these fibers penetrated into the presubplate zone, invaded the deep portion of the CP, and participated in SP formation. Fibers of both the ESS, originating from the basal forebrain, and prospective thalamic fibers from the ISS participated in an invasion of the deep portion of the CP and SP formation. After this morphogenetic event, the expanded synaptic SP becomes the closest cortical compartment to the ESS, which was clearly visible on sections stained with AChE (Fig. 3d, arrow). Expansion of the SP from the deep loose portion of the CP (Kostović and Rakić 1990; Duque et al. 2016) appeared to ‘‘create’’ more space for the ingrowth of SS fibers, as discernable on in vivo MRI scans showing SP formation (Fig. 3g).

### Midgestation (15–22 PCW): spread of fibers from the ESS and ISS to all neocortical regions and the establishment of a new multilaminar axonal-cellular compartment (MACC)

**Fig. 4:**
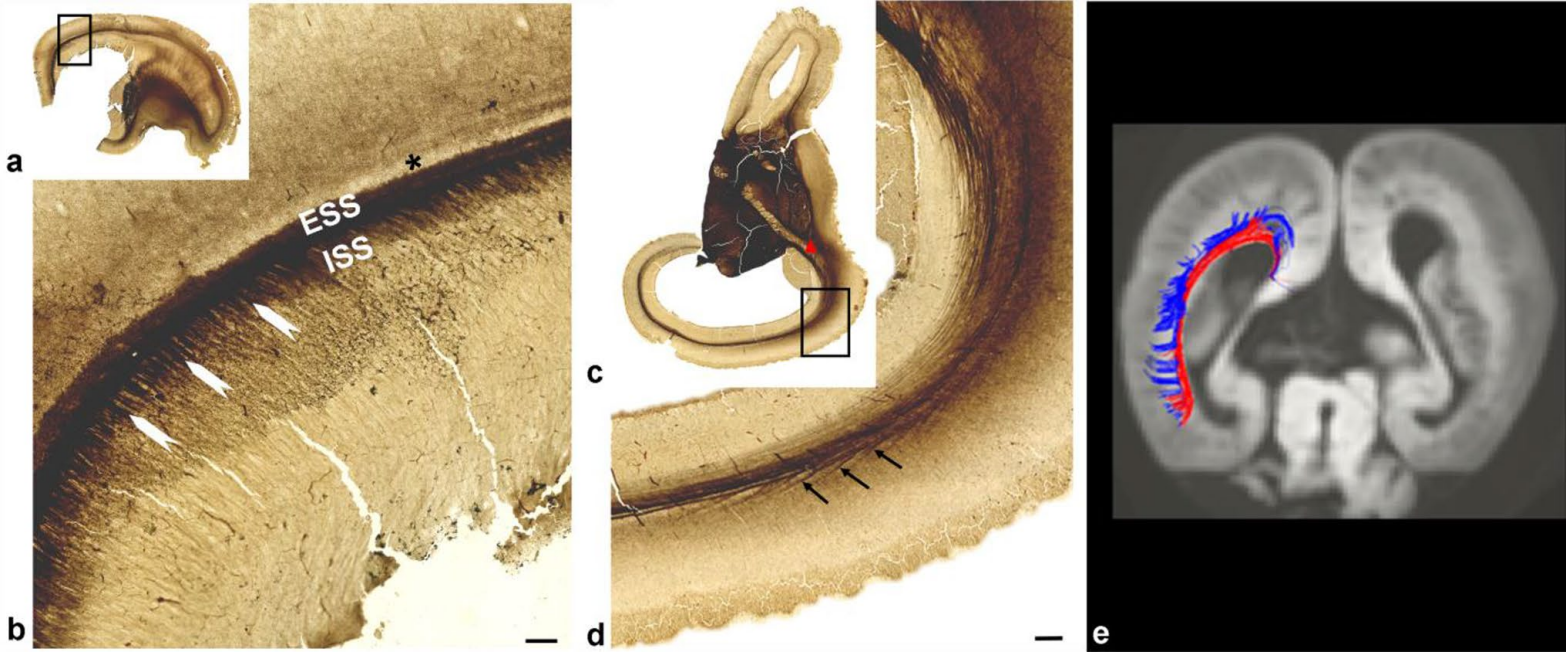
Histological sections of a 16 PCW old fetal brain stained for AChE and cut in sagittal (**a, b**) and horizontal (**c, d**) planes. Rectangles in **a, c** are shown at higher magnifications in **b, d**. Sagittally running AChE-reactive fibers of internal (**b** ISS) and external (**b** ESS) sagittal stratum intersect with radially oriented fibers to form radial “striations” and grid-like patterns (**b** arrowheads). AChE-unreactive band on the outer border of the external capsule (**b** asterix) represents the prospective associative pathways. The oblique course of fibers in the axonal sagittal strata became apparent when approaching the cortex (**d** arrows). Red triangle **c** represents point where two separate AChE-stained pathways merge together forming sagittal strata and run further in continuity towards occipital pole. Scale bar = 250 µm. DTI of 17 PCW old brain shows callosal fibers running in the subventricular zone (red), and callosal fibers running on the border subventricular/intermediate zone (blue), intersected with other sagittal fibers, showing continuity of callosal fibers around ventricles. Scale bar = 250 µm

**Fig. 5:**
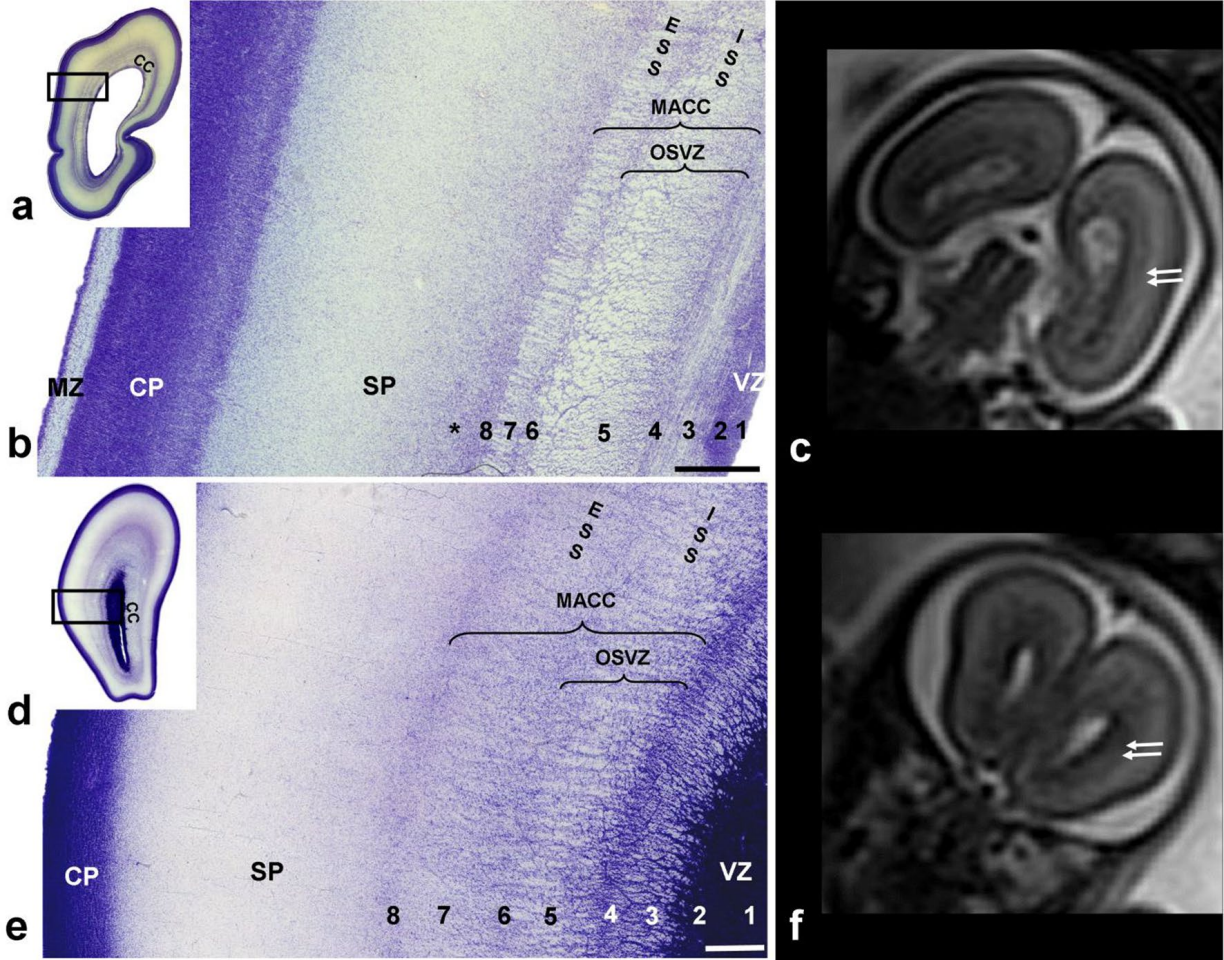
Multilaminated structure of fibrillar and cellular strata in the subventricular zone (SVZ) and intermediate zone, shown on Nissl-stained sections through the occipital (**a, b**) and frontal (**d, e**) lobe of 21 PCW old brain. Rectangles in **a, d** are shown at higher magnification in **b, e**. Laminas under numbers 1–8 represent: (1) ventricular zone; (2) inner subventricular zone; (3) periventricular (callosal) fiber-rich zone; (4–6) complex fibrillar/cellular stratum composed of the outer subventricular zone (OSVZ) mixed with fibers of the ISS composed of an inner (proliferative) cell layer (4); bulk of sagittal fibers (5) which disperse cells from the OSVZ; (6) outer (proliferative) cell layer; (7) external sagittal stratum with strictly packed thalamocortical projection fibers. External (adjacent) to number 7 runs external capsule, but its demonstration requires AChE staining (see Figs. 7, 8c); (8) external transient (proliferative) cell band. The asterisk (**b**) represents cell-free lamina which corresponds to prospective associative fibers. This stratified cyto- and fiber- architectonic organization can be also seen in earlier stages, as 15 PCW (not shown). Hypointense bands on T2-haste MRI (**c, f** double arrows) represent the visibility of sagittal strata on MRI (coronal plane) through the occipital (c) and frontal (f) lobe of a 22 PCW brain scanned in utero (*VZ* ventricular zone, *OSVZ* outer subventricular zone, *MACC* multilaminar axonal-cellular compartment, *SP* subplate, *CP* cortical plate, *MZ* marginal zone, *ESS* external sagittal stratum, *ISS* internal sagittal stratum, *CC* corpus callosum). Scale bar = 500 µm

During midgestation, thalamic fibers situated in the ISS, and basal forebrain fibers situated in the ESS, grew tangentially and longitudinally in a sagittal direction towards the frontal and occipital pole (Fig. 4). Separation of the ISS and ESS was clearly visible at the exit of the posterior limb of the IC (PLIC; Fig. 4c, triangle). SS fibers, when approaching the SP, showed an oblique course (Fig. 4d, arrows). The ISS ran in close proximity to the SVZ (Fig. 4d), where AChE-reactive fibers showed loose arrangement. ESS fibers showed two types of fiber arrangement: more superficial fibers were strongly stained with AChE and correspond to the EC, while deeper running fibers were interrupted by radially oriented striations which were not reactive to AChE staining (Fig. 4b, arrowheads). A very interesting band was visible on the outer border of the EC which was not reactive to AChE staining (Fig. 4b, asterisk). During midgestation, the spatial relationship between SS fibers, migratory cell strands, and the glial elements became more complex, particularly in the occipital lobe, and this clearly defined and organized cellular–fibrillar architecture remains present in the later period, around 22 PCW (Fig. 5b, e).

**Fig. 6:**
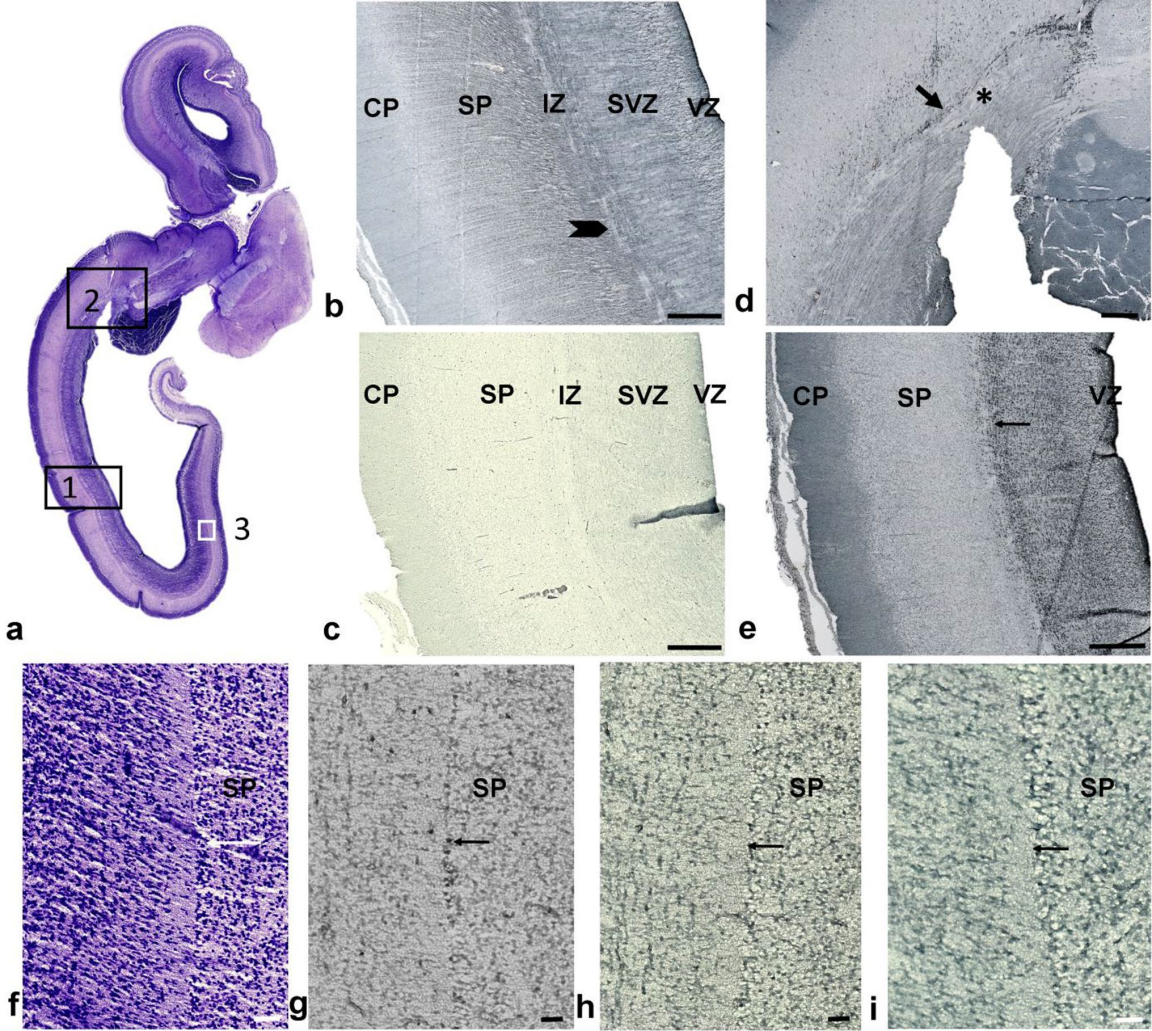
Relationship of the axonal sagittal strata (SS) with glial precursors and glia during midgestation shown on horizontal sections from a 15 PCW brain. Rectangles in **a** (Nissl-stained section) denote approximate locations shown at higher magnifications on sequential sections stained with different markers, as follows: 1 (**b, c, e**); 2 (**d**); 3 (**f**–**i**). GFAP staining (**b**) showed radial glia fibers transversing the subventricular (SVZ) and intermediate zone (IZ) which continued in radial form into the subplate (SP). Bundles of axons (**b** arrowhead) ran sagittally between tightly packed radial glia. The microglial marker CD45 (**d**) was concentrated along the roots of the axonal SS, to the so-called, crossroads (**d** asterisk), and did not show a laminar distribution, except in the very origin of the axonal SS, close to the crossroads (**d** arrow). OLIG2-reactive cells (**c**) were evenly distributed in the SS and did not show a predominant laminar distribution. In contrast to the non-laminar distribution of OLIG2, PAX6-positive radial glia (**e**) showed good correlation with Nissl-stained laminar organization (see Fig. 6b). PAX6-positive cells were concentrated in the outer proliferative cell layer (**e** arrow; marked as lamina ‘6’ on Fig. 5b). Mediooccipital portions of the external proliferative cell band (**f**–**i** arrow; marked as lamina ‘8’ on Fig. 5b), showed nonspecific affinity for certain stainings (**f** Nissl; **g** CD45; **h** Iba1; **i** PAX6), where this narrow “corridor” cell layer created a sharp boundary towards the overlying SP. Scale bar (**b**–**e**) = 500 µm; (**f**–**i**) = 50 µm

During this period, the ISS intermingled with cells of the OSVZ (terminology introduced by Smart 2002), while the ESS intermingled with outer/external transient cell bands. In general, compartments containing SS fibers were very cellular. The multiple alternation of fibrillar strata and cellular layers leads to profound transformation of the OSVZ and deep IZ, which then jointly formed a new compartment in the cerebral wall. This compartment showed a very characteristic multilaminar organization on Nissl-stained sections (Fig. 5b) and was composed of fibrillar (axonal) and cell layers. We believe that the most appropriate term for this compartment is the “multilaminar axonal-cell compartment” (MACC). The cell density in this new compartment was higher than in the early IZ and the abundance of fibers was more prominent than in early SVZ. The cytoarchitectonics and fibrillar arrangement (Fig. 5b) of the MACC in the occipital lobe was geometrically well defined. Sharp delineation of the occipital ESS (Fig. 5b, marked with 7) was very characteristic and precisely spatially ordered: fiber bundles within this stratum were separated by strands of cells which merged at the external and internal sides of the stratum, with a tangentially oriented outer proliferative cell layer (Fig. 5b, marked with 6) and an external proliferative transient cell band (Fig. 5b, e, marked with 8). Radially oriented strands and sagittally running fibers give this stratum a palisade-like appearance (Smart et al. 2002; Zecevic et al. 2005), corresponding to the outer fibrillar layer, described by Smart et al. (2002) (OFL, discussed later). Thus, the layers of the deep periventricular compartment were defined as follows: (1) ventricular zone; (2) inner SVZ (ISVZ); (3) PVFZ; (4–6) complex cellular/fibrillar stratum composed of OSVZ mixed with fibers of the ISS containing an inner (proliferative) cell layer (4) together with bulk of sagittal fibers (5) which dispersed cells of the OSVZ, and outer (proliferative) cell layer (6); ESS (7) with thalamocortical projection fibers and an outermost fiber stratum (EC) containing fibers from basal forebrain and (8) external (proliferative) transient cell band (Fig. 5b, e). Prospective associative fibers develop along the deep border with the SP (Fig. 5b, asterisk). On in vivo MRI scans, not all layers were visible and well delineated. On Fig. 5c, f, one can see a single T2 hypointense ventricular zone, the PVFZ, and a single T2 hypointense band which probably corresponds to the MACC of the histological section.

**Fig. 7:**
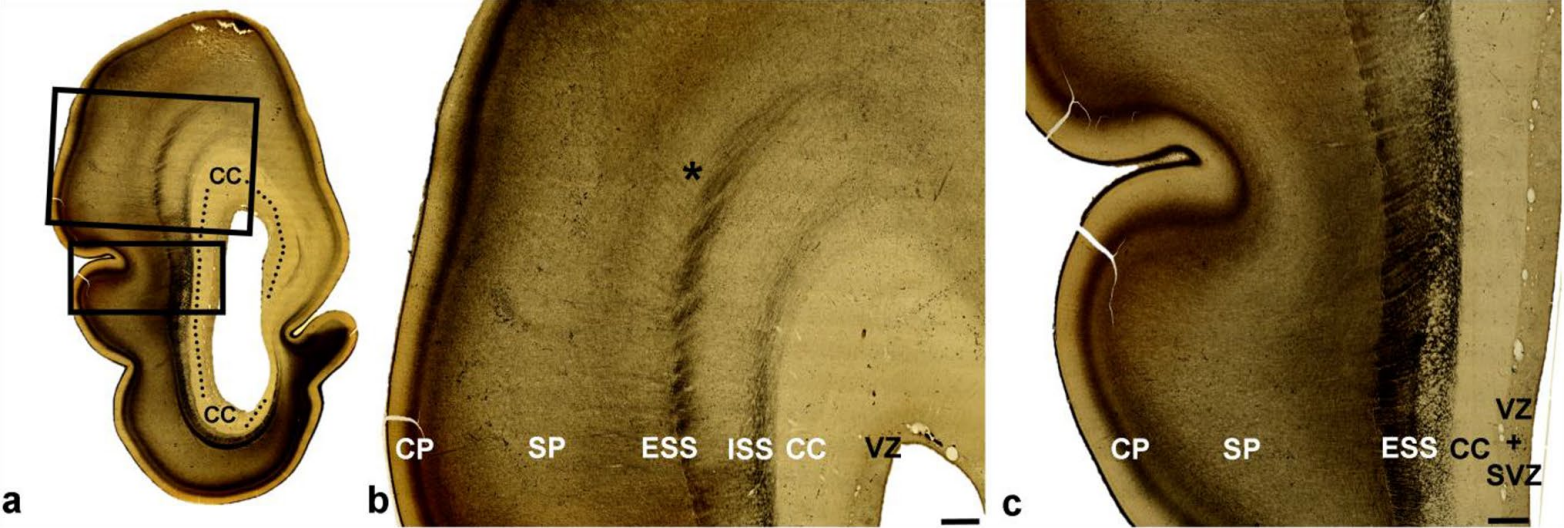
Coronal section through the parietooccipital part of a brain at 26 PCW and stained for AChE showing pulvinocortical and basal forebrain fibers of axonal sagittal strata as AChE-reactive. Callosal (CC) and associative (**b** asterisk) fibers, and primary optic radiation are AChE-unreactive. CC is characterized by continuity around ventricles (**a** dots). Rectangles in **a** are displayed at higher magnification in **b, c**. (*VZ* ventricular zone, *SVZ* subventricular zone, *ISS* internal sagittal stratum, *ESS* external sagittal stratum, *SP* subplate, *CP* cortical plate). Scale bar = 1 mm

**Fig. 8:**
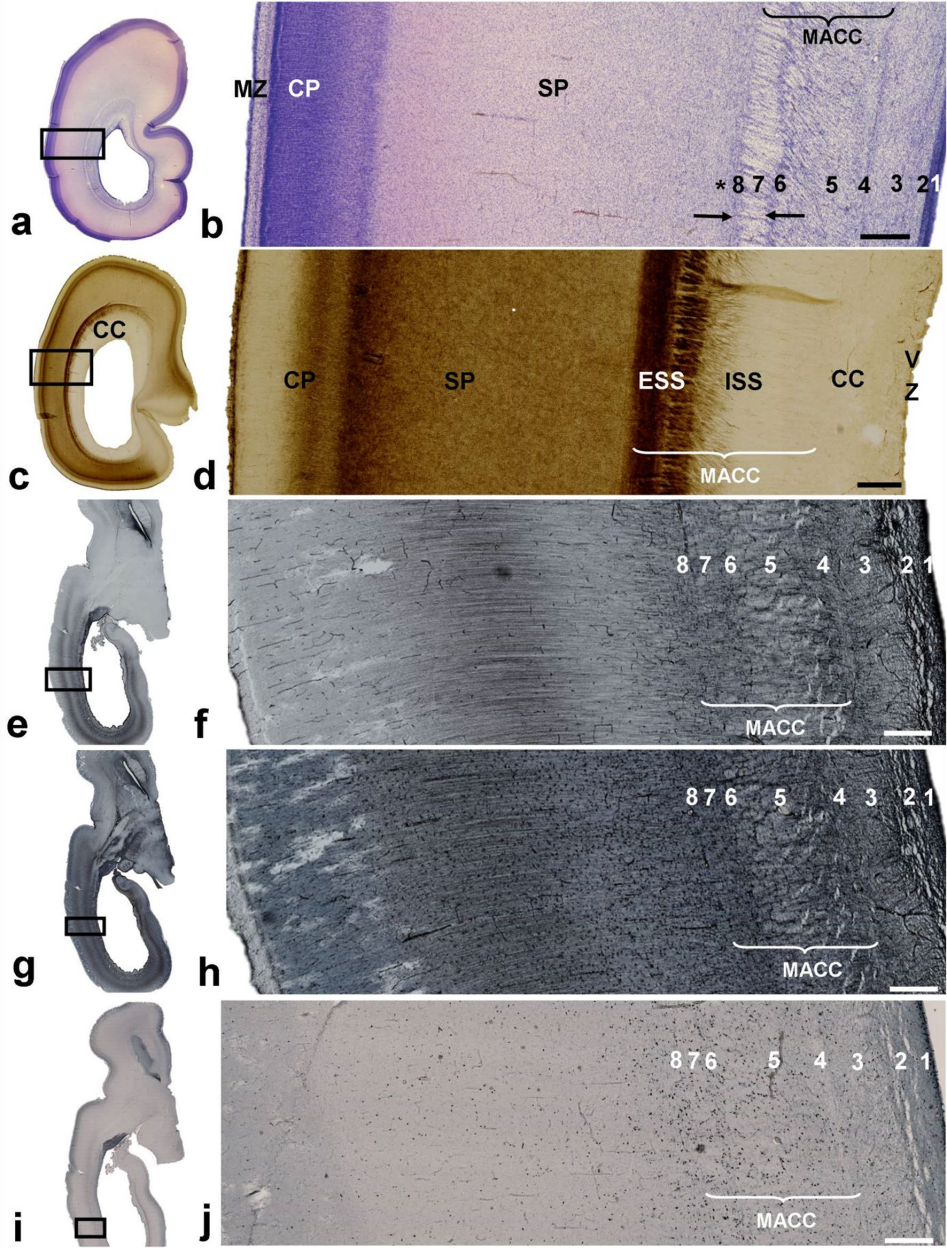
Laminar organization of the sagittal strata during the early preterm period. Coronal Nissl-stained frozen section (**a, b**) through the parietooccipital portion of the brain at 26 PCW showing a similar laminar organization as seen in the previous phases (see Fig. 5). The internal sagittal stratum (ISS) is densely cellular (**b** number 5), and this stratum may be partially defined on both AChE-stained and unstained portion (**d**) on section of the brain at the same age. At this age, both cellular (O)SVZ and former fibrillar intermediate zone now are incorporated in multilaminar axonal-cellular compartment (MACC). Rectangles in **a, c, e, g**, and **i** are shown at higher magnifications in **b, d, f, h**, and **j**, respectively. Sublamination of the sagittal fiber strata and related cell layers were also visible on horizontal histological sections of brain at 24.5 PCW when immunostained for vimentin (**e, f**), GFAP (**g, h**), and Ki67 (**i, j**). Numbers (1–8) used to mark the laminas are explained in detail in Fig. 5. Immunostaining for glial markers (**e**–**h**) showed that the radial glia transverses the radial–sagittal strata and subplate (SP) to reach the cortical plate (CP). Immunostaining for proliferative markers (**i, j**) showed proliferative activity in cellular–fibrillar layers, except in the compact periventricular (callosal) system. The two proliferative cell layers (**b** between arrows) were not a constant finding on Nissl-stained preparations of the occipital lobe (see Fig. 5). Asterisk (**b**) represents prospective associative fibers. Radial striations were visible both on Nissl and AChE preparations due to the intersection of radially oriented cell strands and sagittally running fibers (*VZ* ventricular zone, *CC* corpus callosum, *MZ* marginal zone). Scale bar = 500 µm

Comparison between cytoarchitectonically defined layers and sublayers of the MACC, and the distribution of various glial markers, showed partial compatibility, where the closest compatibility was shown by the prospective radial glia marker PAX6 (Fig. 6e), which showed accumulation in the outer proliferative cell layer (Fig. 6e; arrow; also marked as number 6 on Fig. 5b). Distinct monolayer corresponding to the external transient cell band in medial and distal portions of the occipital lobe (Fig. 6f–i, arrow) shows precise alignment along the growth “corridor” of the ESS and led us to refer to these cells as “corridor cells”. GFAP staining showed stronger immunoreactivity in the ESS, where radial fibers intersect with sagittal-running fibers, leaving fiber-rich zones unstained (Fig. 6b, arrowhead). The microglial marker, CD45, showed alignment of microglia along the roots of the SS (Fig. 6d, arrow) where the SS emanated from the crossroad area (Fig. 6d, asterisk). In contrast to these findings, the oligodendroglial marker, OLIG2 (Fig. 6c), was distributed throughout the axonal SS without laminar preference.

In the developing frontal lobe (Fig. 5d, e), the arrangement of SS fibers and proliferative cellular zones was different and geometrically less strictly defined than in the occipital lobe. The first difference was in the periventricular fiber-rich (callosal) zone (PVFZ). Specifically, massive callosal fibers in the developing genu of corpori callosi surrounded the developing anterior horn of lateral ventricles from the medial, dorsal, and lateral sides. The PVFZ corresponding to the inner fibrillar layer (IFL) described by Smart et al. (2002), was intersected with closely spaced striations composed of cellular elements, previously described as the callosal septa (Jovanov-Milosevic et al. 2009). In the occipital lobe, periventricular callosal fibers formed a sagittally oriented thinner sheet along the lateral wall of the ventricles (fore-runner of the tapetum) but avoided the calcarine area. Furthermore, thalamocortical and cortico-subcortical fibers of the ISS were less compact and almost completely dispersed in the OSVZ (Fig. 5e).

### Period corresponding to extremely early preterms (22–28 PCW)

During this period, sagittally running fibers, originating from the thalamus and basal forebrain, accumulated in the superficial SP, below the CP, and gradually penetrated this cell dense layer. The AChE-reactive fibers of the thalamus and basal forebrain, which ‘‘exit’’ from the axonal SS, ran in oblique short trajectories before entering the SP. In the basal portion of the occipital lobe, SS fibers were more compact, and the ESS was close to the ISS (Fig. 7c). In the dorsal portion of the parietooccipital part of brain, the SS is wider as a consequence of widely spaced ISS and ESS (Fig. 7b). The exact arrangement of different classes of projection, commissural, and associative fibers within the SS was difficult to determine on regular histological sections stained with Nissl, but this arrangement was more obvious on preparations stained with AChE. The position of AChE-reactive fibers from the pulvinar (Kostović and Rakić 1984) and basal forebrain (Kostović 1986) was visualized with histochemical AChE staining (Fig. 7). AChE-unreactive commissural CC bordered the medial side of the ISS and OSVZ. It was situated along the roof of the ventricle, was of a large size, formed a ‘‘callosal plate’’, and was easily identified (Fig. 7a, CC). Fibers from the pulvinar occupied a medial position within the occipital SS, while fibers from the basal forebrain formed the EC, within the most external part of the SS (Kostović and Rakić 1984). Research has shown that primary visual projection is well developed by that time (Hevner 2000; Vasung et al. 2017), but this is not reactive to AChE staining during the early preterm period, had already formed a three-dimensional loop (Kostović and Rakić 1984; Krsnik et al. 2017), and represents a prominent component of the SS. The position of efferent, corticofugal fibers within the axonal SS is difficult to determine without tracing methods. In the brains of experimental animals, it has been shown that efferent fibers occupy a deeper position in ‘‘fetal’’ white matter (Bicknese et al. 1994; Molnár et al. 1998). This corresponds to the classical rules (Sachs 1892), which state that, during development, long, earlier-developing, fiber systems are closer to the ventricles, while later-developing short pathways are closer to cortex. Corticofugal fibers were not reactive to AChE staining and obviously correspond to AChE-unreactive zones within the SS. They also develop relatively early; corticospinal fibers were found to reach decussation level in the brainstem by 15 PCW and the lower spinal cord by 24 PCW (Eyre et al. 2000; Eyre 2003; Staudt 2007), while corticopontine fibers are an early component of the periventricular fiber system (Vasung et al. 2011). In the superior part of the occipital lobe, there is a narrow zone of AChE unreactivity (Fig. 7b, asterisk), separating the EC and deeper parts of the SP. This zone marks the prospective development of associative fiber bundles.

**Fig. 9:**
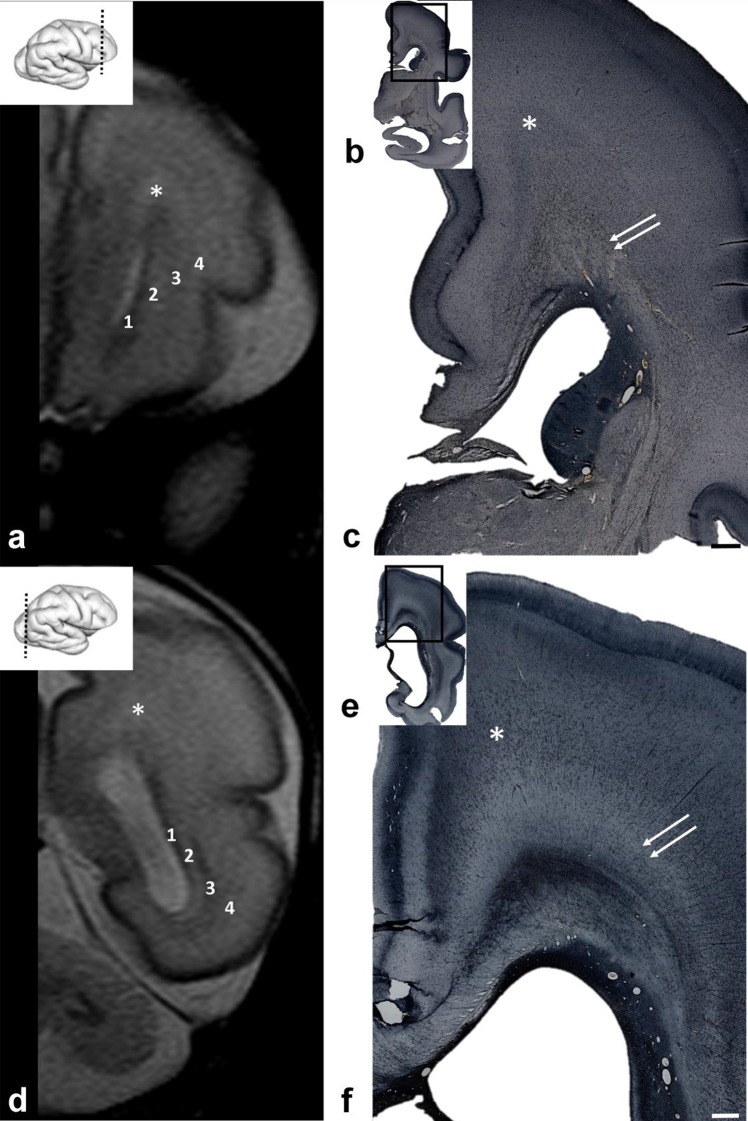
In vivo T2-weighted MRI scans (coronal plane) through frontal (**a**) and occipital (**d**) parts of the brain of an infant born prematurely and scanned at 27 PCW showing a T2 hypointense band (**a, d** number 3) which marks the positions of the sagittal strata and associated cell layers. This band is delineated on the pial side by a hyperintense signal at the subplate. Towards the ventricle, the narrow lamina of higher MR intensity (**a, d** number 2) separates this band from the hypointense ventricular zone (**a, d** number 1). There was clear microglial accumulation along the sagittal strata (**c** double arrows) and crossroad (**c** asterisk) on coronal sections taken through a 27 PCW brain immunostained for Iba1. Rectangles in **b, e** are shown at higher magnifications in **c, f**. The two components of the white matter second segment, sagittal strata and the crossroad (marked with an asterisk on **a, c, d, f**), show lower concentrations of GFAP-positive elements (**f**) on coronal sections through the occipital part of the same brain as in Fig. 9 c. Double arrows (**c, f**) highlight the transition of the sagittal strata into the crossroad (**f** asterisk). A clearly apparent T2 hypointense band (**a, d** number 3), mostly corresponding to the external proliferative transient band when comparing to histological sections, and it represents border to T2 hyperintense lamina which actually does not stand alone but merges with large T2 hypointense subplate (**a, d** number 4). External proliferative layer gradually disappears in later stages (see Fig. 12). Scale bar = 1 mm

In general, the multilaminar organization of cell layers and fiber strata (Fig. 8) resembles the organization seen in the midgestational brain in that the VZ is reduced in size, but the OSVZ and ISS remain very cellular. During this period, the distribution of glial and proliferative markers (Fig. 8e–j), and the distribution of migratory neurons, also resemble midgestational organization, where fiber-rich strata alternate with cell-rich zones. Cell strands in the ESS are less pronounced, but this part of the SS still shows a palisade-like appearance. Pallisadic arrangement (radial striations) was visible both on Nissl (Fig. 8b) and AChE staining (Fig. 8d). Proliferative cells were readily found in both the ISS and ESS (Fig. 8i, j), but were rarely seen in the SP. A cell-rich band, situated in the ESS, is more pronounced in the basal, ventral portion of the occipital lobe, and together with a parallel external proliferative layer, appears as two tracks (Fig. 8b, between arrows). The position of this band is an important landmark for visualization of the SS on MRI (Fig. 9a, d, number 3). The stratified appearance of the SS on MRI was enhanced by the differential laminar distribution of glia (Fig. 9b, c, e, f). Microglial distribution (Fig. 9b, c) and GFAP-immunoreactive radial glia (Fig. 9e, f) delineated the crossroad areas (Fig. 9c, f, asterisk; see also Judaš et al. 2005), and these periventricular crossing fibers continue into the SS (Fig. 9c, f double arrow). The spatial distribution of these glial elements enhanced the visualization of layers on MR images (Fig. 9a, d).

### The period corresponding to very preterm and moderate-to-late preterm birth (28–36 PCW): white matter was divided into four segments (I–IV) and all major fiber systems were already present in axonal SS

**Fig. 10:**
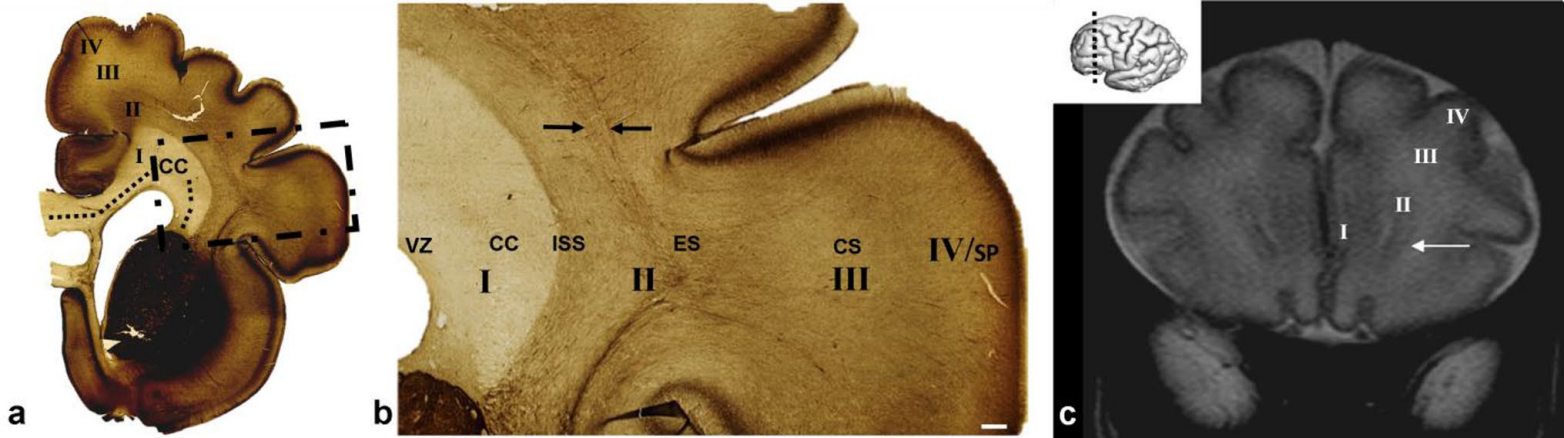
AChE-stained coronal section of a brain at 35 PCW (**a, b**) showing that, in parallel with a reduction in the subplate (SP), the centrum semiovale (CS) develops externally to the external sagittal strata (**b** between arrows; also ESS). In a preterm infant, the architecture of the sagittal strata in the frontal lobe was defined by a massive, non-myelinated, corpus callosum (“callosal plate”, CC) and cellular strands. The rectangle in **a** is displayed at higher magnification in **b**. T2 MRI scans (coronal plane) of the frontal lobe of an infant born prematurely and scanned at a corrected age of 34 PCW, showed a visible cell strand (**c** arrow) as a T2 hypointense band. At that time, two parallel processes are present: gradual resolution of SP together with progressive transformation of fetal white matter into adult type characterized by segmentation into I–V segments according to Von Monakow. CS develops in the core of the cerebral wall due to the massive addition of associative, AChE non-reactive fibers, and is, therefore, less stained. At this stage, segment IV is not fully developed, and it is hard to separate it from SP. Segment V (intracortical white matter) is not shown, because its development occurs postnatally (*VZ* ventricular zone, *ISS* internal sagittal stratum, **a**–**c** I-callosum with periventricular fibers; II-crossroads and sagittal strata; III-centrum semiovale; IV/SP-gyral white matter/SP). Scale bar = 1 mm

**Fig. 11:**
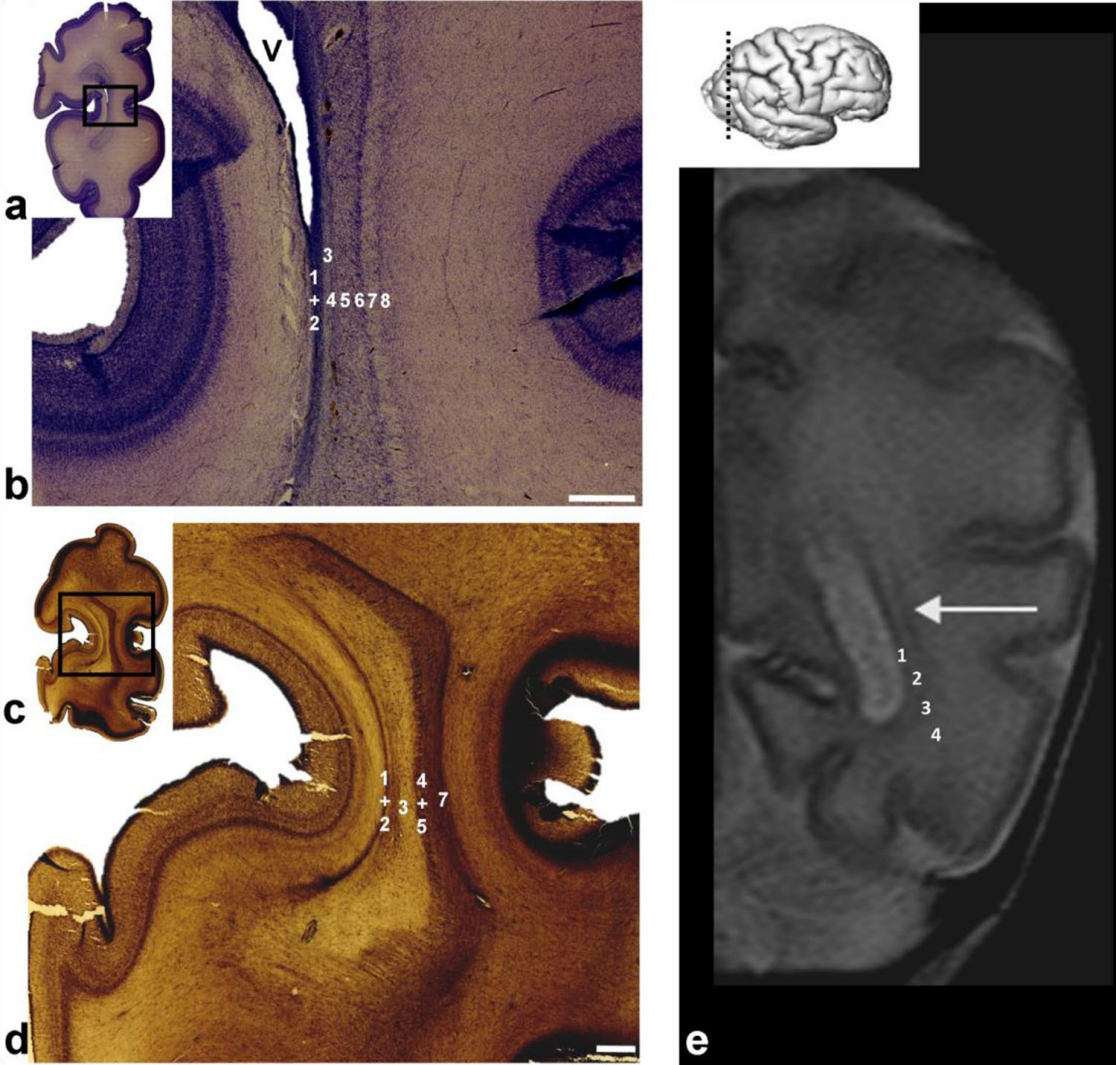
Nissl (**a, b**) and AChE-stained (**c, d**) coronal sections through the occipital lobe of a brain at 35 PCW showing a two-track appearance as the main hallmark of sagittal strata organization. Rectangles in **a, c** are shown at higher magnification in **b, d**. The laminar structure (**b, d**) resembled the same organization as in the previous stage (see explanation of numbers 1–8 on Fig. 5), only with less striations in the fiber-rich zone (**b** marked with ‘7’), compared to earlier stage. T2-weighted MRI scan in the coronal plane of the occipital part of a preterm infant brain, born at 31 PCW, and scanned at 34 PCW (**e**) shows clearly apparent alternating layers of differing signal intensity on T2-weighted images consisting of alternating hypointense (numbers 1, 3) and hyperintense (number 2, 4) laminas, signifying the position of the sagittal strata (explained in detail in Fig. 9). Two T2 hypointense bands surround the T2 hyperintense lamina to form a characteristic “triplet” structure. Important: numbers on histological sections do not represent the same structures as those on MRI scans; *V* ventricle. Scale bar = 1 mm

The rapid development of distal white matter segments III and IV (centrum semiovale and gyral white matter; Fig. 10) is primarily related to the development of the associative fiber system (Vasung et al. 2010, 2017; Mitter et al. 2015). In parallel with this process, there is a reduction of SP thickness (Kostović and Rakić 1990) and a reduction in SP volume (Vasung et al. 2016). The lateral delineation of SS towards SP is now significantly changed, in particular in frontal lobe (Fig. 10a, b, CS), due to the development of centrum semiovale (Fig. 10b, III). Centrum semiovale is composed of massive associative cortico-cortical fibers, which intermingle with other projection pathways. Associative fibers are AChE-unreactive, and, therefore, centrum semiovale, which is in the core of cerebral wall is less AChE-stained and stands out between AChE-stained SP and CP on superficial, and EC on the deep side. An important landmark for medial delineation of axonal SS is the sagittal extensions of the CC. A large cross-sectional area of the CC (Fig. 10a, b) stood out on sections stained with AChE, because callosal fibers are AChE-unreactive, which is in contrast to AChE-reactive SS fibers. AChE-reactive fibers originating from the thalamus and the basal forebrain within the occipital and frontal SS form three discrete “tracks”: the internal “track” which borders with the CC and corresponds to the ISS, and two external “tracks” which correspond to the ESS. A comparison between sections stained with Nissl (Fig. 11a, b) and AChE (Fig. 11c, d) showed that transient cell layers, seen on Nissl staining, were partially in congruence with fibers which were reactive to AChE staining. However, no complete compatibility was evident, since AChE-reactive fibers are just one component of the SS. The most reliable identification of all fiber systems is for the PVFZ: these fibers are not AChE-reactive and continue into the massive callosal system at genu and splenial levels. The voluminous PVFZ of the preterm brain may also be delineated on preparations stained with Nissl by virtue of a pale, cell-poor appearance. Transient cell bands were still present along the outer and inner borders of the ESS; the external transient cell band on the outer border (number 8 on Fig. 11b) and a parallel-running cell layer on the inner surface of the ESS (number 6 on Fig. 11b), together with the inner cell layer of the SVZ (number 4 on Fig. 11b), contain proliferative cells, which are important constituents of transient layers and participate in stratification of the occipital SS. However, the overall number of cells which were immunoreactive for SOX2 was reduced compared to the extremely preterm period (not shown). Despite a reduction in the number of proliferative cells, transient cell bands remain as an important landmark of stratification in the occipital SS of the preterm brain and represent the substrate of the “two-track appearance” on occipital lobe sections stained with Nissl (marked as numbers 6 and 8 on Fig. 11b), and resembled the “two-track appearance” of AChE staining. As stated earlier, these two cell bands actually delineate very conspicuous fibrillar ESS (marked as number 7 on Fig. 11b), which shows fewer radial striations compared to the previous period of development, probably caused by the diminishment of migratory cells, which migrate radially through sagittally oriented fibers of the ESS. The laminar organization of the occipital SS leads to their visibility on in vivo MRI scans (Fig. 11e). The occipital SS appear as alternating layers of differing signal intensity on T2-weighted images. From ventricle to SP, and following differing signal intensity, the following strata were visible: [1] T2 hypointense lamina corresponding to proliferative periventricular zones (Fig. 11e, number 1); [2] T2 hyperintense deep lamina (Fig. 11e, number 2); [3] a T2 hypointense band (Fig. 11e, number 3, arrow); [4] T2 hyperintense lamina which merges with an SP T2 hyperintense compartment (Fig. 11e, number 4). Two T2 hypointense bands surrounded the T2 hyperintense lamina to form a characteristic “triplet” structure (two bands and a central lamina), which could also be identified in all infants born prematurely (aged 26–32 PCW; 13 of 13 examined cases). The signal intensity of the two outer hypointense bands corresponded to the signal intensity of the CP, indicating high packing cellular density, while the central lamina showed signal intensity similar to the white matter. It was difficult to define which microstructural elements visible on Nissl and AChE preparations corresponded to this “triplet” MRI scans, because laminas shown on MRI, due to the limitations of MRI resolution, may include several sublayers, which can only be separated by histological preparations. To be specific, we identified eight sublaminas on Nissl-stained sections during this (Fig. 11b) and earlier developmental stages (Fig. 5), which led us to presume that the MRI “triplet” structure represented the fusion of several cellular and fiber strata. In addition, MRI scans depict the callosal periventricular system (future tapetum) in a variable and inconsistent manner.

### Term age: The MACC was replaced by predominantly fibrillar, deep white matter segments. Delineation of the axonal SS was enhanced by the initial myelination, and had a different appearance in normal newborns born at term when compared to normotypic infants born prematurely and scanned at TEA

**Fig. 12:**
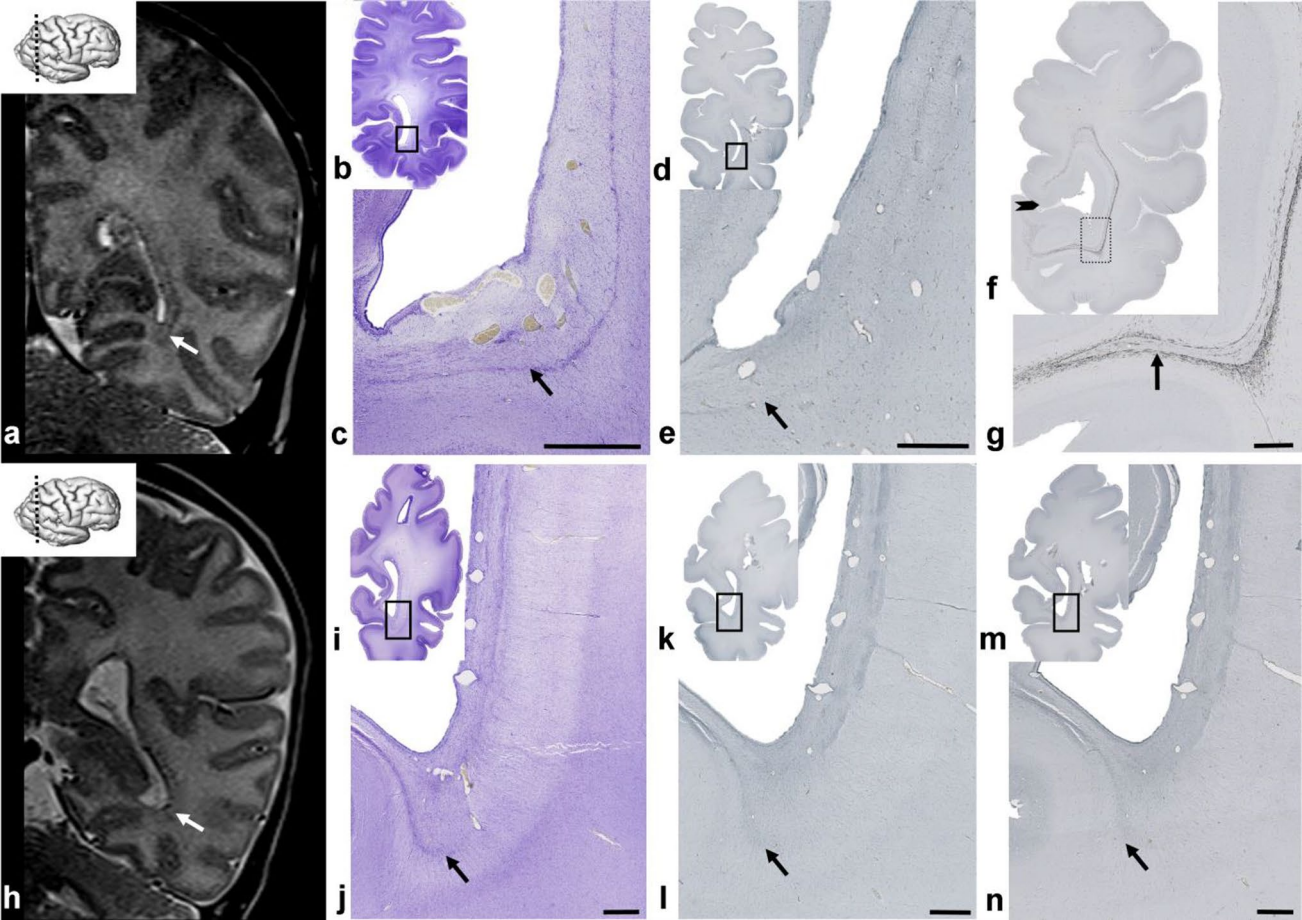
Maturation of the occipital sagittal strata (SS) on coronal plane T2 MR images (**a, h**), and corresponding histological preparations from newborn (**b**–**g**) and preterm (**i**–**n**) brains. The rectangle in **b, d, f, i, k**, and **m** are shown at higher magnification in **c, e, g, j, l**, and **n**, respectively. Comparison of MRI scans through the occipital lobe in a newborn infant (**a**) and a premature infant scanned at term equivalent age (**h**) suggested a slight difference in visibility and delineation of the sagittal strata (**a**, **h** arrow) fashioned as a visible “triplet structure”. Histological sections of Nissl-stained (**b, c; i, j**) preparations, sections immunostained for the proliferative marker Ki67 (**d, e; k, l**) and myelin basic protein SMI99 (**f, g; m, n**) indicated that different underlying factors contribute to the MRI “triplet” appearance in preterm and term periods. The differential prominence of cell and fiber layers, and the differential appearance of proliferative cells and myelination of the visual pathway at preterm and term determined the structure of the occipital SS. The less visible “triplet structure” in a preterm infant at TEA (**h**) was probably due to the disappearance of the transient proliferative cell band (arrow, **l**; please, see MRI scan of preterm brain scanned soon after birth on Fig. 9) and impaired myelination ( **n** arrow). In the newborn brain, histological coronal sections through the occipital lobe (**f, g**), at the level of the calcarine fissure (**f** arrowhead), showed immunoreactivity for SMI 99 in the axonal SS (**g** arrow). Arrow (**c, e, j, l, n**) points out to transient cell band which serves as an example of different maturation processes mentioned above. Scale bar = 1 mm

At term, cellular bands disappeared from the axonal SS and fibrillar component within the MACC, which became more compact rather than its earlier laminated appearance (Fig. 12c). This process was more obvious in the occipital/parietal regions than in the frontal lobe. In the occipital/parietal region, sagittal commissural, projection, and associative fibers ran in parallel, for a long distance along the posterior horn of the lateral ventricles, to form several fibrillar strata. In addition, in the occipital lobe, axonal strata were compressed in dense fiber bundles, and became more compact. The closest stratum to the ventricle is the tapetum; the following stratum streaming from the PLIC, containing thalamocortical radiation, is mixed with efferent pathways. The visual radiation runs within the ventral portion of the ESS. The most superficial are the associative sagittal pathways. The SVZ was no longer visible in sections taken from the frontal lobe due to the massive callosal system, aligned along the thalamocortical and associative fibers, which radiated more superficially and entered the centrum semiovale. Analysis of proliferative markers in the VZ, ISVZ, OSVZ, and SS (Fig. 12e, l) showed resolution of the MACC due to the disappearance of some transient layers, an overall reduction of proliferative activity in the remaining cell layers, and more compact axonal bundles. Only scattered proliferative cells were present in the fiber-rich strata. The VZ and ISVZ were reduced in their extent, and curved along the lateral angle of the anterior horn of the lateral ventricles, forming a “pocket” surrounded by callosal fibers. The VZ, and eventually the SVZ, formed a band along the roof of lateral ventricles, below the transversal portion of the CC and merged with, the so-called, subcallosal zone (Kostović et al. 2002b). In newborns, the appearance of the SS was marked with a new event, the initial myelination of projection fibers, presumably from thalamic nuclei (Fig. 12f, g). At the coronal section of the occipital lobe (Fig. 12f, g), the myelination pattern was very typical; namely, the fibers continuing from the SS, became more compact, forming a more sharply delineated irregular C-shaped trajectory. When approaching the border between areas 17/18, fibers formed a fine plexus in the gyral white matter of area 18 and only a small number of fibers continued into the lower lip of the calcarine fissure. Myelinated fibers in the C-shape sagittal trajectory (Fig. 12f) were very close to the bottom of the calcarine fissure, but did not exit from the stratum and did not enter into the calcarine cortex.

On MRI scans, there were slight differences in appearance of the occipital SS in normal infants born at term and in normotypic infants born prematurely and scanned at TEA. The “triplet” structure was found in all normal term born controls (Fig. 12a) (five out of five cases). On these scans of normal term infants, there was a well-delineated stratum of low T2 signal intensity (Fig. 12a, arrow), which ran parallel to the two strata of high T2 signal intensity. The position and shape of two external tracks within the “triplet” structure corresponded to myelinated stratum, and is marked as an arrow on Fig. 12g. In premature infants, scanned at TEA, SS stratification (the “triplet” structure) was less pronounced on MRI and appeared relatively immature (Fig. 12h). The T2 hypointense band was thin and discontinued and the lower portion of the band was not well delineated (Fig. 12h, arrow). This immature appearance of the “triplet” was found in 8 out of 13 cases (62%). The “triplet” image was missing in two cases (15%) and was poorly delineated in 3 out of 13 cases (23%). Developmental changes on MR scans and in histological structures were best revealed using illustration of landmark phases (Fig. 12). The differences in “triplet” appearance between the occipital SS in preterm infants (Fig. 12a) scanned at TEA and at term (Fig. 12h) were probably caused by three events: an increase in myelination of the visual pathways (compare Fig. 12g, n), the disappearance of transient proliferative bands (compare Fig. 12e, l), and the pronounced compactness of fibers (compare Fig. 12c, j).

## Discussion

The present study revealed several new developmental, structural, and spatial features of axonal SS in the human fetal cerebrum. First, we presented evidence for the developmental continuity of sagittally oriented fibers during sequential growth towards a cortical target area from the early fetal period to term until transformation into segmented white matter of the newborn brain. Second, we documented spatial cohabitation and histogenetic interaction of axonal SS with progenitor cells, migratory neurons, and all types of glial cells and the formation of a new, hitherto not fully understood, MACC during histogenesis of the cerebral wall. Third, we describe the composition, organization, and structural properties of how fibrillar and cellular elements influence the dynamic appearance and changing delineation of axonal SS in the fetal, preterm, and newborn brain.

### Continuity of sagittal fiber orientation, sequential growth of axons, and transformation into radially segmented white matter of the perinatal brain

Sagittally oriented fiber systems of the early fetal cerebral wall are the main constituents of the IZ, defined as cerebral fibrillar compartments (Angevine et al. 1970); (Kostović et al. 2002a; Bystron et al. 2008; Kostović and Judaš 2015). This fibrillar cerebral compartment/zone actually forms sagittally and tangentially oriented “corridors” for bundles of projection axons destined for remote polar portions of the cerebral hemispheres. Within these “corridors”, axonal bundles show stratification (Zecevic and Verney 1995; Del Río et al. 2000; Kostović et al. 2002a), which is probably a result of the combined influence of sequential, time-scheduled growth of different classes of axonal pathways (Kostović and Judaš 2007, 2015; Kostović et al. 2014b), the maintenance of axonal fasciculation through different internal molecular mechanisms and externally available guidance molecules (Tessier-Lavigne 1992; Judaš et al. 2003; Charron 2005). Cellular and molecular mechanisms governing growth through sagittal “corridors” of the IZ are largely unexplored in the human brain. However, our current data show that borders (walls) of “corridors” for axonal growth contain characteristically aligned cells (“corridor” cells), which express some of the glial markers and may provide structural and molecular support for axonal growth through the axonal SS towards target polar areas. In addition, our data indicate that there is a structural, spatial (“compartmental”) framework for axonal growth, in addition to the molecular mechanisms described in the previous experimental literature (Tessier-Lavigne 1992), which exists from the early life and determines the laminar position of the main afferent system in IZ; thalamic afferents occupy intermediate and deep positions in the IZ (Kostović and Goldman-Rakić 1983; Kostović and Rakić 1984; Vasung et al. 2010, 2017; Krsnik et al. 2017), while basal forebrain afferents form (more superficial) the ESS which is in the continuity with the external capsule (Kostović and Rakić 1984; Kostović 1986; Kostović et al. 2002a). This presumably cholinergic projection was also previously identified and visualized in the adult human brain (Selden 1998). In the present study, we further confirm the advantages of using transient AChE histochemical staining to study the growth of thalamocortical and basal forebrain fibers (Kostović and Goldman-Rakić 1983; Kostović and Rakić 1984; Kostović 1986; Kostović and Judaš 2010). However, in the present study, we also study other transient chemical features of the fiber-rich IZ, such as the early immunostaining for SNAP25 and synaptophysin. Synaptophysin was detected in the fibers of the IZ, which is devoid of synapses (Molliver et al. 1973; Kostović and Rakić 1990), indicating that synaptophysin reactivity in the IZ and the SVZ is not, at that specific time, related to synaptic distribution (not shown). Transient staining of fibrillar zones using synaptic markers was also described in human fetal material by Bayatti et al. (2008) and Sarnat (2013). Such histochemical and immunocytochemical techniques cannot, however, detect cortical long-efferent projection pathways, which also develop during comparative phases of fetal development (Eyre et al. 2000; Eyre 2003; Staudt 2007), after the first pioneer projection (Meyer et al. 2000) has already established. The continuity of sagittally running (projection, commissural, and associative) fibers within the common fibrillar “corridor” raises the question of developmental sequence and time overlap in respect of the growth of different fiber systems. The sequential ingrowth of afferent fiber systems, observed in the present study, is in accordance with the previous studies of the developing human brain (Marin-Padilla 1970; Nobin and Björklund 1973; Olson et al. 1973; Mrzljak et al. 1988; Zecevic and Verney 1995; Kostović et al. 2002a; Judaš et al. 2005; Kostović and Judaš 2007). The first efferent pioneer projection originating from calretinin-reactive cells of the pioneer neurons (Meyer et al. 2000) also takes a course within the IZ. However, the first robust fiber system constituting the IZ and the SS is projection fibers from the thalamus (Molliver et al. 1973; Kostović and Goldman-Rakić 1983; Kostović and Rakić 1984; Kostović and Judaš 2010; Krsnik et al. 2017) and efferent cortical fibers (Eyre et al. 2000; Vasung et al. 2010, 2011), as well as projection fibers from the basal forebrain (Kostović 1986). The early development of thalamocortical fibers is consistent with our observation relating to the early dispersion of the SVZ. Within the thalamocortical projection system, the early development of primary visual projection from the lateral geniculate body was first demonstrated by Hevner (2000) and more recently by Vasung et al. (2017), and projection from pulvinar was documented by Kostović and Rakić (1984). The callosal system (Ren et al. 2006) runs periventriculary in the PVFZ (Kostović et al. 2002a; Vasung et al. 2011). The fiber system within the deep SVZ was also described as inner fibrillar layer (IFL) by Smart et al. 2002, using histological sections prepared from the primate brain, although not specifically attributed to the CC, and it was marked correctly on sections from the Yakovlev collection (Rakić and Yakovlev 1968; Bayer and Altman 1991; Wang et al. 2015). According to the anatomical description of the adult human brain presented in the classical literature, callosal fibers, which form forceps in both an anterior and posterior direction, run for a considerable distance around the ventricles in sagittal directions, to form the tapetum in the occipital lobe (Sachs 1892; Déjerine 1895; Von Monakow 1905; Polyak 1957; Hosoya et al. 1998; Schmahmann and Pandya 2006). In the present study, we found that sagittally running callosal fibers markedly delineate the ISS on the medial side of the occipital lobe, gradually invading the PVFZ and SVZ around 15 PCW. More precisely, callosal fibers split the deepest portion of the SVZ, adjacent to the VZ. The occipital extension of the CC in PVFZ appears to be more pronounced in the human brain compared to other species and seems to be absent in the rodent brain (Smart et al. 2002).

There is a general agreement that long associative pathways for the lateral aspect of hemispheres develop relatively late in fetal life (Catani et al. 2003; Huang et al. 2006; Kostović and Jovanov-Milošević 2006; Kasprian et al. 2008; Vasung et al. 2010, 2017; Takahashi et al. 2012). However, evidence from a previous MR tractographic study supports the idea that the main trajectory of all long associative pathways is visible by birth (Kasprian et al. 2008; Vasung et al. 2017). Of all associative pathways, the inferior frontoocciptal fascicle (iFOF) shows the closest association with the SS. Other associative pathways are lateral or more ventral from the SS. Indeed, the iFOF develops in the lower (ventral) extension of the EC, and has been described as an associative pathway (Takahashi et al. 2012; Huang and Vasung 2014; Mitter et al. 2015). We have clearly demonstrated that the main portion of the EC, and its radiation, contains projection fibers from the cholinergic basal forebrain (Kostović 1986) and develops at least two months earlier than the associative pathways. Within the ESS, the associative iFOF develops later within the EC showing the same fan-like radiation as the primordial EC.

### Multilaminar arrangements and the changing appearance and incorporation of axonal strata into a new axonal-cellular compartment reflect basic histogenetic processes in the cerebral wall: ingrowth of axonal pathways, proliferation, migration, cell aggregation, delamination of fetal zones, and myelination

Our results indicate that, at around 15 PCW, the new MACC forms at the interface between the IZ and the SVZ. In this new transient compartment of the cerebral wall, axonal SS, derived from the IZ (Judaš et al. 2005; Bystron et al. 2008; Kostović and Judaš 2015), alternate with several layers of neuronal progenitor cells, glia, post-mitotic tangentially and radially migratory neurons derived from the SVZ (Levitt et al. 1981; Kostović and Rakić 1990; Smart et al. 2002; Zecevic et al. 2005; Bystron et al. 2008; González Gómez and Meyer 2014). In the late fetal phase, there is a massive ingrowth of long associative fibers, which grow along the EC (Huang et al. 2006; Kasprian et al. 2008; Vasung et al. 2017) and contributes to the multilaminar complexity of this dynamic axonal-cellular compartment. After invasion of the SVZ and the formation of a prominent SP, between 13 and 15 PCW (Kostović and Rakić 1990; Duque et al. 2016), growing sagittal fibers within the MACC become strategically positioned between the synaptic SP (Molliver et al. 1973; Kostović and Rakić 1990) and the proliferative zones, and may participate in a major neurogenetic event, the intensive production of cortical neurons from intermediate progenitors (Reillo et al. 2011; De Juan Romero and Borrell 2015; Dehay et al. 2015; Nowakowski et al. 2016; Popovitchenko and Rasin 2017). In addition, these fibers can play a role in gliogenesis (Rakić 1981; Zecevic et al. 2005; Nowakowski et al. 2016) and neuronal migration (Rakić 1981). Our results strongly suggest that the process of morphogenetic interaction between the growing axonal SS and the proliferative compartments occurs in several steps. First, projection fibers from the thalamus initially disperse the SVZ (as early as 11 PCW). During the next phase (12.5–15 PCW), projection pathways cause multiple stratification of the SVZ, while callosal fibers delaminate the VZ–ISVZ from the OSVZ. After 15 PCW, the MACC is formed, and is composed of axonal SS derived by transformation of the IZ alternating with proliferative cell layers derived from the SVZ. In the early preterm period, the characteristic composition of the MACC is still very prominent. External to the MACC, associative fibers form an additional fibrillar stratum. During subsequent preterm, projection, commissural, and associative axons of the axonal SS converge in the centrum semiovale, parallel with the formation of gyral white matter (Kostović et al. 2002a, 2014a). At term, a reduction of proliferation activity and an increase in compactness and myelination of the axonal SS become important markers of white matter integrity. The fact that we observed strong proliferative cells incorporated in the MACC is not surprising, because we also detected massive invasion of the SVZ by main axonal projection pathways and transformation into the MACC. Our identification of the MACC sheds new light on the structure of the proliferative compartments. The close association of proliferative compartments with fiber systems was previously described in humans (Kostović et al. 2002a; Zecevic et al. 2005; Bayatti et al. 2008; Molnár and Clowry 2012) and monkeys (Dehay et al. 2001; Smart et al. 2002; Reillo and Borrell 2012). The importance of callosal fibers, as main constituents of PVFZ, corresponding to the internal fibrillar layer is emphasized in other parts of discussion. We have already described another component of periventricular fiber system, fasciculus frontooccipitalis, in close proximity with the proliferative ganglionic eminence, indicating possible role in proliferation control and interaction with periventricular proliferative structures (Vasung et al. 2011). The relationship between sagittal fibers and the OSVZ is even more interesting, while the SVZ is known as an essential proliferative compartment of the cerebral wall in primates (Rakić 1988, 1981; Smart et al. 2002; Lukaszewicz et al. 2005; Bystron et al. 2008; Howard et al. 2008; Clowry et al. 2010; Hansen et al. 2010; Reillo et al. 2011; De Juan Romero and Borrell 2015; Dehay et al. 2015; Kostović and Judaš 2015; Nowakowski et al. 2016). The fact that we observed massive invasion of the SVZ and its transformation into the MACC offers a comprehensive interpretation of proliferative compartments in the large primate brain and will eventually help to overcome the previous inconsistencies in the existing literature relating to delineation description, terminology, and the interpretation of cellular and fibrillar laminas of the cerebral wall in the late fetus (Kostović and Rakić 1990; Bayer and Altman 1991; Smart et al. 2002; Bystron et al. 2008; Nowakowski et al. 2016).

The presence of proliferative markers (Ki67) in the MACC indicates the importance of this phenomenon, both for developmental and diagnostic purposes. From the developmental point of view, the close relationship between axons and neuroepithelial progenitor cells is interesting due to the fact that DNA synthesis in proliferative progenitor cells of the VZ and SVZ may be modulated by the non-synaptic release of the neurotransmitters, glutamate, and GABA (LaMantia 1995; LoTurco et al. 1995; Haydar et al. 2000; Kirischuk et al. 2017) and the modulation of calcium-signaling (Spitzer 2006). The significance of a diffusible factor that promotes the proliferation of cortical precursors, and attributed to thalamic axons, was described by Dehay et al. (2001, 2015) and also proposed by both Molnár and Clowry (2012) and Reillo and Borell (2012). Our present data extent this intriguing role for other fibers in the axonal SS; the massive ingrowth of fibers in the axonal SS may predominantly exert mechanical influence and displace progenitor cells from their signaling environment and secondarily, affect proliferative activities. In addition, massive axonal ingrowth may have an indirect effect on SVZ proliferative layers by providing factors for dispersion/pushing apart progenitor and post-mitotic cells to change the extracellular micro-environment (Polleux et al. 2001) and thus diminish availability and efficiency of signaling molecules (Dehay et al. 2015).

It is very likely that developmental interaction between axonal and glial elements and migratory neurons facilitates function in terms of the growth of axons within corridors and early interactions with migratory neurons. In this context, the most interesting point to note is the transient cell band (marked as ‘8’ on our figures) which is located next to external border of the MACC, adjacent to the EC. This, most external, cell band of the MACC continues around the curvature of the occipital lobe, reaching the medial side of the hemisphere, where it becomes visible as a monolayer; this markedly defines the border between projection fibers of the SS and the SP (Kostović and Rakić 1990). This cell band shows variation across the hemisphere in terms of both compactness and thickness and its appearance in the medial ventral portion of the developing occipital lobe corresponds to the transient cell band illustrated by Hoerder-Suabedissen and Molnár (2015). Cell accumulation in this layer is the prospective substrate for the visualization of the axonal SS in preterm infants on MRI. This transitory phenomenon, and cell band, disappears by birth and can, therefore, no longer be used as a structural substrate for the visualization of the axonal SS. The presence and variable appearance of this band in the preterm brain may serve as an additional marker of the intensity and variability of neurogenetic processes in the human brain. Transient appearance of the axonal SS with notable individual differences (Sachs 1892), variable stratification of proliferative zones, waves of migratory neurons, and the curved appearance of late radial glia with intermingling fibers during the late fetal period may also be responsible for variable histological delineation of the subventricular proliferative compartment in the primate brain (Rakić 1981; Bystron et al. 2008; Nowakowski et al. 2016) and thus cause contradictory interpretation of radial organization in the late human cerebral wall. The massive tangential fiber bundles of the axonal SS may also result in the discontinuous appearance of the radial glia on histological sections (Nowakowski et al. 2016) and thus mask the actual presence of continuity in the radial glia scaffold during the late phases of corticoneurogenesis. The radial orientation of glial fibers and tangential orientation of the axonal SS form a grid-like framework, which is necessary for the maintenance of crucial neurogenetic processes, migration (Rakić 1981, 1988), and axonal growth.

The fact that we observed dynamic changes in both cellular composition and appearance indicates that the “triplet” appearance formed by different MRI signal intensity bands is caused by variation in the relative contribution of different elements in preterm brain (this study) than in pediatric or adult brain (Hosoya et al. 1998; Wichmann and Müller-Forell 2006; Naidich et al. 2013).

### Differences in the appearance of axonal SS between the preterm and newborn brain is influenced by differential composition, organization, and structural properties of fibrillar and cellular elements

To evaluate the usefulness of the normative data presented in the present study in terms of the developmental interpretation of fetal white matter lesions, it is first necessary to classify cellular layers and the axonal SS on the basis of histological and MRI correlation (Kostović et al. 2002a; Huang and Vasung 2014; Wang et al. 2015); second, to relate this classification to the current neurobiological terminology. Thus, we will first compare our histological-MRI classification of cerebral compartments with currently accepted terminology (Bayer and Altman 1991; Kostović et al. 2002a; Smart et al. 2002; Jakovcevski and Zecevic 2005; Bayatti et al. 2008; Bystron et al. 2008; Borrell and Reillo 2012; Lewitus et al. 2013; Wang et al. 2015; Dehay et al. 2015; Nowakowski et al. 2016) and the classical description of the axonal SS (Sachs 1892; Von Monakow 1905; Brodmann 1914; Polyak 1932; Schmahmann and Pandya 2006).

Based on our present data, the proposals of different research groups, and comparisons between current and classical terminology, we offer a new histologically and histogenetically grounded classification for cellular and fibrillar strata. There is a general agreement that the cell layer, which encompasses ventricles and contains neuroepithelial stem cells and neuronal progenitors, is the VZ (Hevner 2006; Bystron et al. 2008; Borrell and Reillo 2012; Dehay et al. 2015; Nowakowski et al. 2016; Popovitchenko and Rasin 2017). However, there is less agreement with regards to the nature and origin of the fiber-rich layer, which is present in dorsolateral portions of cerebral hemispheres, splitting the SVZ into the ISVZ and the OSVZ. This layer, designated as IFL (Smart et al. 2002; Zecevic et al. 2005; Molnár and Clowry 2012; Reillo and Borrell 2012; Dehay et al. 2015; Wang et al. 2015; Nowakowski et al. 2016), was described by Bayer and Altman (1991) as STF6, and as PVFZ by Kostović et al. (2002a). The continuity of this zone with the CC was mentioned in the previous paragraphs of this paper. There is no controversy in terms of the ISVZ, which is simply a narrow portion of the SVZ adjacent to the VZ (Smart et al. 2002). Since the ISVZ is narrow and irregularly serrated, and the SVZ is not split into two parts in the early fetal life, the classical description considered these structures as one entity (Bystron et al. 2008). However, the current literature focuses more on a prominent OSVZ, as described in the monkey brain by Smart et al. (2002). This terminology was accepted in subsequent papers, probably because this represents an important prominent proliferative zone of the primate brain (Hansen et al. 2010; Lewitus et al. 2013; Dehay et al. 2015; Nowakowski et al. 2016). Today, this term is generally accepted and it may not be feasible to change terminology. However, our results may show that the robust ingrowth of fibers from the ISS disperse previously the cytoarchitectonically uniform SVZ during midgestation and subsequent stages to form a new transient MACC. The appearance of the MACC, which contains alternating stratification of massive fibers and proliferative cell layers, shows that the major volume of the SVZ is transformed, making it difficult to define exactly which part of the proliferative compartment should be referred to as the OSVZ. We have described this MACC as a structure composed of proliferative cells and fiber-rich axonal SS, showing developmental continuity with the early fetal IZ. We emphasize that the formation of this complex layer during midgestation, and its existence during late gestation, is an important histogenetic event which facilitates interaction between the growing fiber system and the proliferative compartments of the large human brain.

The outer fibrillar layer (OFL; Smart et al. 2002; Dehay et al. 2015) is definitely the ESS, and, in the occipital lobe, contains fibers of optic radiation (Sachs 1892; Polyak 1957; Hosoya et al. 1998; Wichmann and Müller-Forell 2006; Naidich et al. 2013). Given that this may also contain other fibers system in its dorsal aspects, we intend to refer to the ESS as previously described in the classical literature. For example, our current data and previous observations indicate (Kostović 1986; Kostović et al. 2002a; Vasung et al. 2010) that the outermost fiber system in the ESS of the preterm brain belongs to the radiation of the EC, accompanied by associative fibers of the iFOF and eventually the inferior longitudinal fascicle (Catani et al. 2003). Thus, the OFL, as a complex fiber system composed of thalamic and basal forebrain fibers in earlier stages, and associative fibers in subsequent development, is better described as ESS, and in earlier stages, as an external portion of the IZ. Because of the complex composition of the ESS, with a minimum of three fiber systems (thalamocortical, basal forebrain and associative), we are more inclined to use classical terminology (Sachs 1892; Von Monakow 1905). The changing picture of multilaminar organization in the SVZ and IZ highlights the difficulty in selecting universal terminology for the large, slow-developing human brain where the axonal SS are an essential component of the second (II) white matter segment. We consider that, for the early fetal period, term IZ for fibrillar and SVZ for adjacent proliferative zone, as proposed in terminology of updated Boulder Committee (Bystron et al. 2008), is appropriate. For the mid-fetal period, we recommend that we refer to the combination of layers proposed by Smart et al. (2002), with our corrections and our newly identified MACC. We also suggest that the late fetal period requires an adult form of classification and division across different white matter segments (Sachs 1892; Von Monakow 1905; Kostović et al. 2002a, 2014b; Judaš et al. 2005).

Using laminar organization of the axonal SS as a main marker of white matter integrity and a developmental marker during the late preterm and neonatal period, we have found notable developmental differences. In addition, our data suggest a maturational delay in some normotypic infants when compared with MRI scans of normal control cases at term. However, characteristic “triplet” structure of the occipital SS found on MRI scans of preterm babies after birth may show a different microstructure than the “triplet” structure visible at term (the present study), or in the adult brain (Hosoya et al. 1998; Wichmann and Müller-Forell 2006; Naidich et al. 2013). The main argument for this claim is the fact that our histological analysis of preterm SS showed several transient features, which change by the neonatal period. For example, the proliferative layer is associated with ESS but subsequently disappears, and the poor myelination of the fibrillar core of the SS, which becomes myelinated by the time of the neonatal period. In addition, the proliferative VZ and SVZ diminish and gradually disappear (Vasung et al. 2016). Between the late preterm and neonatal period, there is also an increase in the compaction of the callosal fiber system. Thus, myelination, compaction of fiber bundles, and the disappearance of proliferative layers are the main microstructural changes underlying the “triplet” occipital structure of the SS on MRI scans during the neonatal period. There are several possible developmental explanations for this developmental phenomenon. First, at an early age (27–32 PCW) on MRI, a cell dense band is aligned along the ISS. If this cell strand is composed of migratory, (Rakić 1974; Hansen et al. 2010), proliferative, and glial cells, then by birth, this cell band would diminish due to a reduction in proliferative activity and the end of migration (Rakić 1974). The explanation for the sharper delineation of the axonal SS in normal term brain, compared to an infant born prematurely and scanned at TEA, is the more advanced myelination in the term group. In all “normal” term brains (Zagreb Neuroembryological Collection), we found strongly myelinated fiber bundles running along the VZ and SVZ, and also found that these myelinated tracts stop at the border of visual areas 17/18. This fiber system may represent a projection from the associative pulvinar nucleus, from the visual pulvinar (Kostović and Rakić 1984; Baldwin et al. 2017) and primary visual projection from the lateral geniculate body (Polyak 1957). The other possibility is that the early myelinated fibers belong to geniculocortical projections, as proposed by other authors (Yakovlev and Lecours 1967). At the time of birth, both pulvinocortical and geniculocortical pathways (Kostović and Rakić 1984; Hevner 2000; Guzzetta et al. 2010; Lennartsson et al. 2014; Vasung et al. 2017) are part of the occipital SS. Histological and immunohistochemical evidences for myelination of the optic pathways (Yakovlev and Lecours 1967; Brody et al. 1987; Kinney et al. 1988) are consistent with findings from MRI studies on the early myelination of optic radiation (Barkovich et al. 1988; Battin et al. 1998). However, regardless of whether myelinated fibers in the ESS belong to primary optic radiation or represent a projection from the pulvinar, it is important to note that the myelinated stratum of the SS is an important microstructural component of the SS on MRI scans of newborns. In this context, it is interesting that almost one quarter of premature babies showed a poorly delineated occipital “triplet” structure when scanned at TEA. This finding raises the question of whether the structural appearance of the occipital SS at term may serve as a marker with which to predict outcome following white matter lesions during the preterm period. First, poor delineation of the SS, and in particular, the appearance of “triplet” structure of the occipital SS may represent a marker of myelination delay. The delay of SS myelination in premature infants may require rather complex explanations. However, the most plausible explanation is that premature birth, in combination with hypoxic ischemia, causes ‘‘lesions’’ in the preoligodendrocytes (Kinney et al. 1988; Back et al. 2001; Volpe et al. 2011). If this interesting explanation is correct, then the fact that we identified proliferative activity within cell bands associated with the adjacent axonal SS may be attributed to the production of preoligodendrocytes destined to form myelin for visual projection pathways. Lesions of these oligodendrocytes may lead to the delayed myelination of fiber constituents in the occipital SS (Back et al. 2001; Volpe et al. 2011). In our earlier paper (Kostović et al. 2014b), we proposed and offered supportive evidence that a ‘‘lesion’’ of individual classes of axons may lead to the enhancement or diminishment of the borders between different segments of white matter on MRI. Here, we propose that the structural appearance of the axonal SS (the enhancement or diminishment of delineation) may represent a reliable marker of white matter integrity (Kostović et al. 2014b). The advantage of investigating the fiber tracks of the occipital SS provides key insight, not only with regards to the projection fibers contained in the PLIC, but also in terms of the integrity of associative and callosal fibers contained in the occipital SS. The presence of associative fibers in the sagittal strata and the adjacent callosal fiber system clearly distinguishes the SS from the PLIC. From a clinical point of view, it is clear that we need to scan more cases at term and at TEA, and carry out careful follow examinations before using appearance of occipital SS on the conventional MRI for the analysis of white matter lesions and as a potential predictive indicator of sensory, motor, cognitive, and behavioural deficit, eventually comparable to currently used MRI criteria (Rutherford et al. 1998; Krägeloh-Mann and Horber 2007; Kidokoro et al. 2011). On the other hand, the normal appearance of deep white matter segments may provide a good prognostic indicator (Kidokoro et al. 2011). We believe that developing knowledge in relation to the normal development of the discrete white matter segments (Kostović et al. 2014b) during the preterm period is crucial for our understanding of perinatal hypoxic–ischemic lesions.
